# The redefined identity of *Prevotella*: new implications for oral, respiratory, and vaginal health

**DOI:** 10.1128/jb.00133-26

**Published:** 2026-07-10

**Authors:** Souzane Ntamubano, De'Jana Parker, Ariangela J. Kozik

**Affiliations:** 1 Department of Molecular, Cellular, and Developmental Biology, University of Michigan 1259, Ann Arbor, Michigan, USA; 2 Division of Pulmonary and Critical Care Medicine, Department of Internal Medicine, University of Michigan 1259, Ann Arbor, Michigan, USA; Dartmouth College Geisel School of Medicine, Hanover, New Hampshire, USA

**Keywords:** taxonomy, microbiome, *Prevotella*

## Abstract

For much of the 20th century, the field of bacteriology was dominated by a pathogen-centric view, which rightly focused scientific resources on identifying, characterizing, and eliminating the agents of infectious disease. However, this perspective has resulted in the relative neglect of abundant yet poorly characterized members of the human microbiota. Few genera embody this oversight more clearly than *Prevotella*. Despite being consistently found in high abundance across diverse human mucosal sites, including the oral cavity, airways, and vagina, the underlying physiological roles of the genus in both health and disease contexts remain largely unclear. This review traces the evolution of *Prevotella* research and classification, arguing that the traditional “friend or foe” dichotomy is insufficient. We contend that more mechanistic work at the species level is needed to elucidate the biology of *Prevotella*, especially after the most recent taxonomic reclassification. The knowledge derived from a mechanistic focus will offer profound benefits for understanding and treating complex polymicrobial diseases across multiple systems, including chronic respiratory infections, oral inflammatory conditions, and recurrent genitourinary dysbiosis.

## BY ANY OTHER NAME: REDEFINING GENUS BOUNDARIES

Over a century ago, Dr. William Wherry and his student, Wade Oliver, reported the isolation of a bacterium they called *Bacterium melaninogenicus* (1). This organism was described as a pigmented, Gram-negative, strictly anaerobic organism found frequently in the human mouth on carious teeth, but also recovered from the throat, tonsils, genitalia, and wound abscesses. Later classified under the genus *Bacteroides*, these and related organisms were frequently described as difficult to grow and maintain in pure culture, with biochemical assays yielding inconsistent fermentation and proteolytic profiles (2, 3). In 1947, Schwabacher, Lucas, and Rimington found that the characteristic pigments were not composed of melanin, as the name suggests, but rather hematin (4). In the 1960s, others showed that some strains required both hemin and vitamin K/vitamin K derivatives for growth (5). Consequently, as similar organisms were isolated from human oral and mucosal sites in the following decades, Shah and Collins proposed the reclassification of several species into their own genus, *Prevotella*, in 1990. Shah and Collins had previously argued that *Bacteroides* contained considerable heterogeneity, particularly with regard to fermentative capacity. They suggested that inclusion in *Bacteroides* should be limited only to highly fermentative species phenotypically similar to *B. fragilis*. The “nonsaccharolytic *Bacteroide*s” were reclassified into the genus *Porphyromonas*, while the remaining 15 “moderately saccharolytic *Bacteriodes*” became *Prevotella*. Using a combination of biochemical and 16S rRNA-based analyses, they described *Prevotella* as organisms that may or may not produce pigment, are sensitive to bile salts, are moderately saccharolytic, and lack both glucose 6-phosphate dehydrogenase (G6PDH) and 6-phosphogluconate dehydrogenase (6PGDH) (6).

Over the next two decades, the acceleration of human microbiome studies contributed significantly to our knowledge of *Prevotellaceae* diversity. By 2021, there were 55 characterized and validly named *Prevotella* species with publicly available isolates and genomes, spanning ecological niches from humans to soil. The human-associated species were frequently reported in data sets from oral, blood, airway, gut, and vaginal sites. Beyond cultivation, metagenomic data sets provide a rich source of genome data from which novel species not yet cultured can be detected in metagenome-assembled genomes (MAGs). Pangenome analysis of known species and over 7,000 *Prevotella* assigned MAGs revealed a genus with strikingly broad genetic and ecological diversity and highlighted the extent to which these organisms are understudied (7).

Analysis of subspecies diversity and genomic diversity within *Prevotella* revealed species that are very similar to one another, with slight genomic differences that contribute to their classification as separate species (7, 8). For example, *Prevotella nigrescens* was once classified as a strain of *P. intermedia* but is now recognized as a separate species (9–12). Traditional biochemical assays have played a significant role in this differentiation, with metabolite production serving as an essential method for initial reclassification. The prevailing view in the field is that horizontal gene transfer and genomic plasticity, including mobile elements such as transposons, contribute to the adaptability of *Prevotella* species across diverse human niches (13–15). Their presence from the respiratory tract to the genital tract suggests species-specific differences in metabolic capabilities, oxygen tolerance, and preferences, highlighting their versatility. In a recent preprint, we employed transcriptomic analyses to show that *P. melaninogenica* is not a strict anaerobe but rather can grow in both 2% and 5% oxygen and further possesses oxidative defense mechanisms, including the superoxide reductase desulfoferrodoxin (dfx), which was markedly upregulated under oxygenated conditions (16).

**Fig 1:**
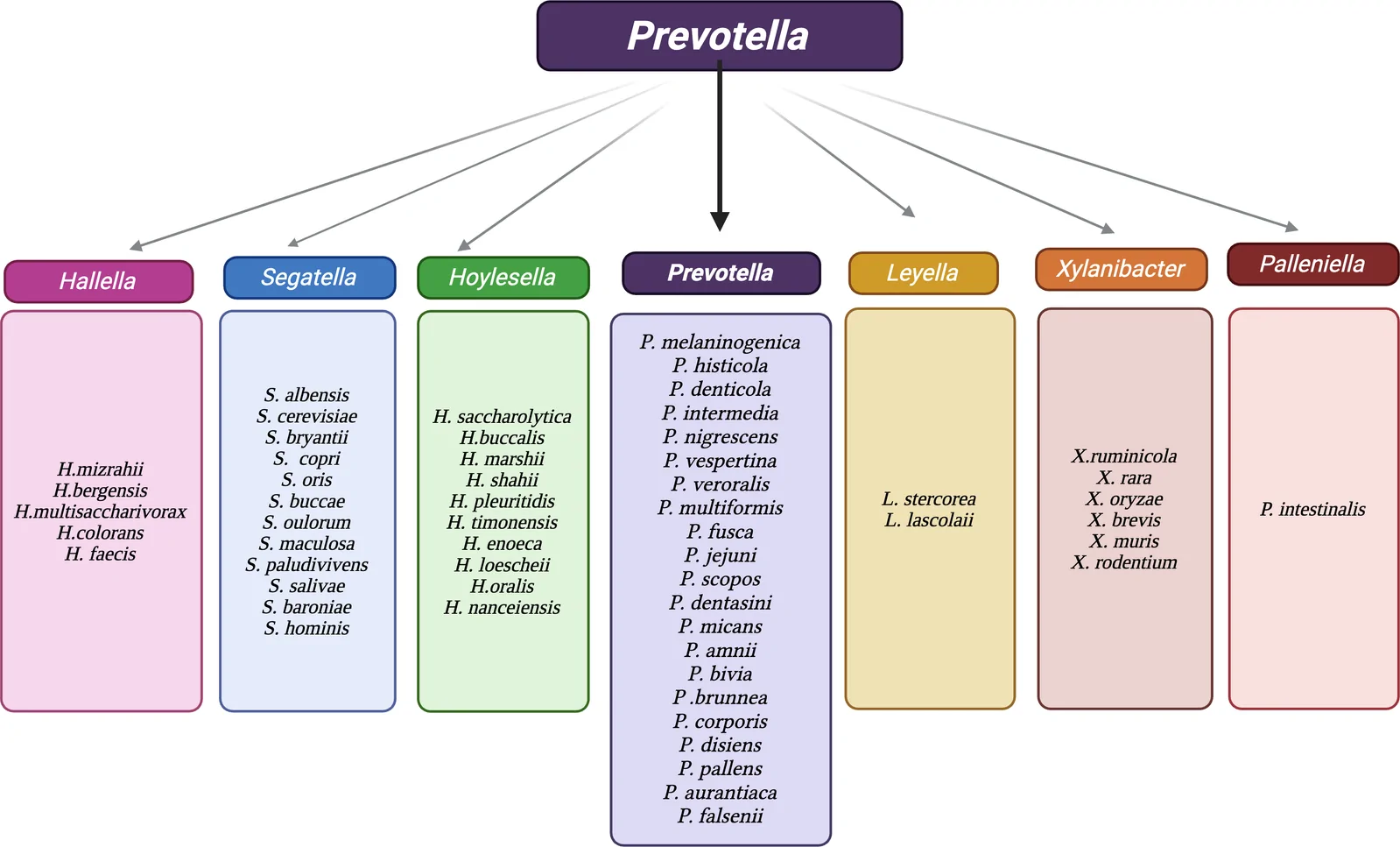
Simplified flow chart of revised and newly added classifications for previously characterized *Prevotella* species. Post-computational analysis resolved seven genera (*Prevotella*, *Hallella*, *Segatella*, *Hoylesella*, *Leyella*, *Xylanibacter*, and *Palleniella*), with 21 of the 55 previously characterized *Prevotella* species still remaining in the *Prevotella* genus. Created with https://BioRender.com.

At the time of writing, our understanding of *Prevotella* has again taken a leap forward. Hitch et al., employing multiple genomic approaches, re-evaluated the relatedness of all known *Prevotella* species. Their analyses showed that these species could be consistently grouped into functionally distinct clades, leading to a reclassification of several *Prevotella* species into different genera. In total, they proposed seven genus-level classifications with amended descriptions of *Prevotella*, *Hallella*, and *Xylanibacter*, and four new genera*—Segatella*, *Hoylesella*, *Leyella*, and *Palleniella*. Out of a large group of 55 species, 21 species now remain classified as *Prevotella* (Fig. 1). These *Prevotella* exhibit shared metabolic (e.g., gene markers) and phylogenomic (e.g., amino acid identity and percentage of conserved proteins) features. *Prevotella* proteomes (with over 90% completeness) were analyzed for phylogenetic distinctiveness using three independent approaches. The first involved constructing a phylogenomic tree using 400 PhyloPhlAn marker genes. Second, species were analyzed using GTDB-Tk (R89) to assign taxonomic classifications based on the bac120 marker gene set. Finally, an Anvi'o phylogenetic tree was generated by identifying 166 of 559 gene clusters using strict functional and structural homogeneity criteria. These phylogenetic trees were then used in further analysis—including average amino acid identity (AAI) comparisons, percentage of conserved protein (POCP) comparisons, carbohydrate-active enzyme (CAZyme)-based profiling, and ecological analysis—to validate the refined taxonomic classifications. This reclassification also proposed the existence of two sub-clades within the *Prevotella* genus (8), although their significance remains to be determined.

Given the extensive use of historically classified *Prevotella* species throughout the microbiome literature, this taxonomic restructuring has important implications for interpreting previous findings. Many studies investigating the ecological, metabolic, and immunological roles of *Prevotella* were conducted prior to the Hitch et al. reclassification and therefore frequently describe species that are no longer considered members of *Prevotella*. Consequently, physiological and pathogenic traits broadly attributed to *Prevotella* may instead reflect the biology of closely related genera now classified as *Segatella*, *Leyella*, *Hoylesella*, *Palleniella*, or *Xylanibacter*. To improve clarity and provide a consistent framework throughout this review, we compiled a comprehensive reference table summarizing the original species classification, updated taxonomic designation, and major biological attributes associated with each historically classified *Prevotella* species discussed in the literature (Table 1; Table S1). Importantly, because much of the foundational literature predates this reclassification, our review frequently considers the broader family *Prevotellaceae* in addition to retained *Prevotella* species. This family-level perspective is particularly relevant for understanding oral, respiratory, and vaginal health, as many of the organisms historically grouped within *Prevotella* occupy overlapping ecological niches, share conserved metabolic capabilities, and exhibit related host interactions despite their revised phylogenetic placement. By explicitly distinguishing retained *Prevotella* species from reclassified *Prevotellaceae* taxa throughout the text, we aim to provide a clearer interpretation of the historical literature while highlighting how this refined taxonomy reshapes our understanding of these organisms in human health and disease.

**TABLE 1:** Summary of historically classified *Prevotella* species including historical nomenclature, current taxonomy, and reported biological attributes described in the primary literature^*a*^

Historical nomenclature	Current taxonomy	Reported biological attributes	References
*Prevotella amnii*	*Prevotella amnii*	Vaginal anaerobic species associated with bacterial vaginosis and adverse reproductive health states; detected in cervicovaginal microbial communities linked to inflammation and altered vaginal microbiome composition; noted as having one of the smallest genomes.	(7, 17, 18)
*Prevotella aurantiaca*	*Prevotella aurantiaca*	Oral anaerobic species associated with polymicrobial biofilm communities and periodontitis; identified in subgingival plaque and shown to possess proteolytic activity and inflammatory potential within oral disease-associated microbial communities.	(19, 20)
*Prevotella bivia*	*Prevotella bivia*	Common vaginal species; associated with BV-related ecology and human infections; co-inoculation with *Gardnerella vaginalis* reproduced BV-like features in mice; recruits CCR5 + TH cells; linked to increased HIV susceptibility.	(7, 17, 21–24)
*Prevotella brunnea*	*Prevotella brunnea*	Anaerobic, pigmented oral bacterium identified within human subgingival and oral microbial communities; associated with periodontitis-associated biofilms and characterized as part of the diverse oral anaerobic microbiota.	(17, 25)
*Prevotella communis*	*Prevotella communis*	Strictly anaerobic saccharolytic bacterium isolated from sheep rumen samples in Slovenia and Japan; specialized in degradation of xylan and pectin-related polysaccharides but unable to utilize starch. Also detected in cattle and sheep rumen metagenomes from Scotland and New Zealand.	(17, 26)
*Prevotella corporis*	*Prevotella corporis*	Anaerobic Gram-negative bacterium originally isolated from the human oral cavity and associated with periodontal disease and oral polymicrobial biofilms; more recent studies identified the species within gut and vaginal-associated microbial communities linked to host inflammatory environments.	(7, 17, 27–29)
*Prevotella dentalis*	*Prevotella dentalis*	Anaerobic Gram-negative bacterium isolated from human oral and dental infections, including periodontal and endodontic sites; associated with oral polymicrobial biofilms and characterized through phenotypic and genomic analyses as a distinct oral *Prevotella* species.	(7, 8, 17, 30–32)
*Prevotella dentasini*	*Prevotella dentasini*	Anaerobic bacterium isolated from human dental plaque and oral clinical specimens; associated with periodontal disease-associated microbial communities and further characterized through genomic analyses examining oral *Prevotella* diversity and taxonomy.	(8, 17, 33, 34)
*Prevotella denticola*	*Prevotella denticola*	Anaerobic oral bacterium isolated from human dental and periodontal sites; identified within polymicrobial oral biofilms and characterized through genomic and phylogenetic analyses examining oral *Prevotella* diversity and taxonomy.	(7, 8, 17, 35–37)
*Prevotella disiens*	*Prevotella disiens*	Anaerobic bacterium identified in human vaginal and urogenital microbial communities, particularly in association with bacterial vaginosis and dysbiosis; studies also reported its presence in polymicrobial infections and linked the species to inflammatory vaginal environments and altered microbial community structure.	(7, 38, 39)
*Prevotella falsenii*	*Prevotella falsenii*	Originally isolated from monkey dental plaque and described as a *Prevotella* intermedia-like anaerobic species based on phenotypic and 16S rRNA analyses. Later studies identified *P. falsenii* in gut microbiome data sets associated with DSS-induced colitis and in fecal microbiota analyses examining functional constipation, depression, and anxiety following fecal microbiota transplantation.	(8, 17, 40–42)
*Prevotella fusca*	*Prevotella fusca*	Anaerobic species isolated from the human oral cavity and differentiated from closely related oral *Prevotella* species using phenotypic and 16S rRNA analyses. Comparative pan-genome analysis later identified *P. fusca* among oral *Prevotella* species associated with periodontitis and characterized its genomic repertoire related to virulence and adaptation within periodontal niches.	(17, 43, 44)
*Prevotella herbatica*	*Prevotella herbatica*	Anaerobic bacterium isolated from a methanogenic reactor treating cattle farm waste; characterized as capable of decomposing plant polysaccharides, including xylan and pectin-derived substrates, and producing acetate and succinate as major fermentation products.	(17, 45)
*Prevotella histicola*	*Prevotella histicola*	Anaerobic Gram-negative bacterium originally isolated from the human gastrointestinal tract. Studies reported that oral administration of *P. histicola* modulated immune responses in multiple sclerosis and arthritis models, including expansion of *Allobaculum* and increased butyrate production. In mice, *P. histicola* reduced LPS-induced olfactory bulb inflammation associated with rhinitis. In cystic fibrosis bronchial epithelial cells, the species activated alternative NF-κB signaling pathways distinct from those induced by *Pseudomonas aeruginosa*. A complete genome sequence for strain T05-04 was also reported.	(7, 8, 17, 46–50)
*Prevotella ihumii*	*Prevotella ihumii*	Anaerobic bacterium isolated from human stool specimens and initially described as a novel species from a healthy woman. Subsequent studies identified *P. ihumii* in gut microbiome analyses associated with cirrhosis and impaired inhibitory control, while genomic characterization studies examined Cas12a nuclease diversity among closely related bacterial orthologs including *P. ihumii*.	(17, 51–54)
*Prevotella illustrans*	*Prevotella illustrans*	Anaerobic Gram-negative bacterium isolated from human oropharyngeal abscess puncture fluid; characterized using phenotypic, biochemical, and genomic analyses and proposed as a novel species within the genus *Prevotella*.	(17, 55)
*Prevotella intermedia*	*Prevotella intermedia*	Anaerobic oral pathogen frequently detected in periodontal disease, odontogenic infections, and extraoral abscesses. Quantitative molecular studies differentiated *P. intermedia* from *P. nigrescens* in clinical specimens and demonstrated clonal diversity among isolates from diseased and healthy periodontal sites. A recent review further summarized its involvement in periodontal disease and systemic inflammatory conditions, highlighting virulence-associated traits including biofilm formation, proteolytic activity, and host inflammatory modulation.	(7–9, 11, 17, 28, 56–60)
*Prevotella jejuni*	*Prevotella jejuni*	Anaerobic Gram-negative bacterium originally isolated from the jejunum of a child with celiac disease and characterized as a novel *Prevotella* species using phenotypic and 16S rRNA analyses. A later metagenome-genome-wide association study detected *P. jejuni* within oral microbiome data sets and identified associations between oral microbial composition and host genetic loci influencing salivary and tongue dorsum microbiota profiles.	(17, 61, 62)
*Prevotella koreensis*	*Prevotella koreensis*	Anaerobic Gram-negative bacterium isolated from human subgingival dental plaque collected from a periodontitis lesion; characterized using phenotypic, biochemical, chemotaxonomic, and genomic analyses and proposed as a novel species within the genus *Prevotella*.	(17, 63)
*Prevotella lacticifex*	*Prevotella lacticifex*	Anaerobic ruminal bacterium characterized as a lactate-producing member of the rumen microbiota with carbohydrate fermentation capabilities. A later structural equation modeling study identified associations between rumen bacterial abundance, including *P. lacticifex*, and lactating dairy cow host phenotypes related to production and physiological traits.	(17, 64, 65)
*Prevotella marseillensis*	*Prevotella marseillensis*	Anaerobic Gram-negative bacterium isolated from the stool of a patient with recurrent *Clostridium difficile* infection	(17, 66)
*Prevotella melaninogenica*	*Prevotella melaninogenica*	Pigmented anaerobic Gram-negative bacterium associated with oral and respiratory environments. Recent studies characterized its starch utilization locus and carbohydrate metabolism using a defined basal medium assay system. In cystic fibrosis lung polymicrobial communities, *P. aeruginosa* supported *P. melaninogenica* survival through metabolic cross-feeding interactions. Another study reported enrichment of *P. melaninogenica* in the lower respiratory tract of patients with checkpoint inhibitor pneumonitis and radiation pneumonitis.	(7, 8, 17, 67–69)
*Prevotella merdae*	*Prevotella merdae*	Anaerobic Gram-negative bacterium isolated from human feces and characterized as a novel species using phenotypic, biochemical, and genomic analyses. A later clinical study reported associations between gut microbiota composition, including *P. merdae*, and improved responses to anti-PD-1 immunotherapy following fecal microbiota transplantation in patients with refractory metastatic solid cancers.	(17, 70, 71)
*Prevotella micans*	*Prevotella micans*	Anaerobic Gram-negative bacterium isolated from the human oral cavity and described as a novel species using phenotypic and 16S rRNA analyses. A later study investigating tryptophanase-mediated indole production in *Prevotella* identified molecular mechanisms contributing to indole biosynthesis among oral *Prevotella* species, including *P. micans*.	(7, 8, 17, 72, 73)
*Prevotella multiformis*	*Prevotella multiformis*	Anaerobic Gram-negative bacterium isolated from human subgingival plaque	(17, 74)
*Prevotella nigrescens*	*Prevotella nigrescens*	Anaerobic oral bacterium associated with periodontal disease, odontogenic infections, and extraoral abscesses. Molecular studies differentiated *P. nigrescens* from *P. intermedia* in clinical specimens and demonstrated clonal variation among isolates recovered from healthy and diseased periodontal sites.	(7, 8, 17, 57–59)
*Prevotella pallens*	*Prevotella pallens*	Pigmented anaerobic bacterium proposed as a novel species isolated from the human oral cavity and differentiated from related oral *Prevotella* species through phylogenetic and phenotypic analyses. Subsequent studies examined β-lactamase production among *P. pallens* genotypes and evaluated *in vitro* susceptibility to antimicrobial agents commonly used against anaerobic oral pathogens.	(7, 8, 17, 75, 76)
*Prevotella pectinovora*	*Prevotella pectinovora*	Anaerobic bacterium characterized as a novel pectin-degrading species based on phenotypic and genomic analyses; capable of utilizing pectin and other plant-derived polysaccharides, supporting a role in carbohydrate degradation.	(7, 17, 77)
*Prevotella phocaeensis*	*Prevotella phocaeensis*	Anaerobic Gram-negative rod isolated from the stool of a patient with *Clostridium difficile* infection during a culturomics study of the human gut microbiota. The species was characterized using phenotypic testing, MALDI-TOF MS, and noncontiguous finished genome sequencing and proposed as a novel species within the genus *Prevotella*.	(8, 17, 78)
*Prevotella rectalis*	*Prevotella rectalis*	Strictly anaerobic Gram-negative rod isolated from a human rectal sample obtained during a culturomics study. The species was characterized by MALDI-TOF MS, phenotypic and biochemical testing, fatty acid analysis, and whole-genome sequencing, which supported its designation as a novel species within the genus *Prevotella*.	(17, 79)
*Prevotella scopos*	*Prevotella scopos*	Anaerobic bacterium isolated from the human oral cavity and described as a novel species using phenotypic characterization and 16S rRNA gene analyses alongside *Prevotella fusca*.	(7, 17, 44)
*Prevotella veroralis*	*Prevotella veroralis*	Anaerobic oral bacterium identified as the causative organism in a rare lung abscess case diagnosed using third-generation metagenomic sequencing.	(7, 17, 80)
*Prevotella vespertina*	*Prevotella vespertina*	Anaerobic Gram-negative bacterium isolated from a human abscess in a hospitalized patient and characterized as a novel species using phenotypic, biochemical, MALDI-TOF MS, and genomic analyses.	(7, 17, 81)
*Prevotella copri*	*Segatella copri*	Gut-associated anaerobic bacterium frequently associated with high-fiber diets and complex carbohydrate metabolism; identified as a dominant member of non-Western gut microbiomes with strain-level diversity linked to distinct metabolic and inflammatory phenotypes. Reported in studies examining rheumatoid arthritis, glucose metabolism, host immune modulation, polysaccharide utilization systems, and microbiome-associated disease variability.	(7, 8, 17, 82–90)
*Prevotella oris*	*Segatella oris*	Originally described as *Bacteroides* oris from human periodontitis and other human infections, including oral infections. Later clinical reports identified *P. oris* as a cause of bacteremia and sepsis originating from dentoalveolar abscesses. A subsequent phylogenomic re-evaluation of the genus reassigned the species to *Segatella* based on genomic distinctiveness from retained *Prevotella* species.	(7, 8, 17, 91, 92)
*Prevotella salivae*	*Segatella salivae*	Anaerobic bacterium isolated from the human oral cavity and described as a novel species using phenotypic and 16S rRNA analyses alongside *Prevotella shahii*. A later phylogenomic re-evaluation of the genus reassigned the species to *Segatella*.	(7, 8, 17, 93)
*Prevotella loescheii*	*Hoylesella loescheii*	Anaerobic bacterium reported as the causative agent of a bacteremic skin and soft tissue infection in a human patient, highlighting its potential role in invasive extraoral disease. A later phylogenomic analysis of the genus reassigned *P. loescheii* to the newly established genus *Hoylesella*.	(7, 8, 17, 94)
*Prevotella marshii*	*Hoylesella marshii*	Anaerobic bacterium originally isolated from the human oral cavity and described as a novel species based on phenotypic and 16S rRNA analyses. A later clinical case report identified *H. marshii* as the causative organism in a pleural infection. Subsequent phylogenomic analysis reassigned the species from *Prevotella* to the genus *Hoylesella*.	(8, 95, 96)
*Prevotella oralis*	*Hoylesella oralis*	Originally described as *Bacteroides oralis*, an anaerobic bacterium isolated from the human oral cavity. The species was later reassigned to the genus *Prevotella* during broader taxonomic restructuring of pigmented anaerobic oral bacteria. Subsequent phylogenomic analysis further reassigned the species to the genus *Hoylesella* based on genomic and phylogenetic distinctiveness from retained *Prevotella* species.	(7, 8, 17, 97, 98)
*Prevotella nanceiensis*	*Hoylesella nanceiensis*	Anaerobic bacterium originally isolated from human clinical specimens and characterized as a novel *Prevotella* species using phenotypic and molecular analyses. A later phylogenomic study reassigned the species to the genus *Hoylesella*. In cystic fibrosis sputum microbiota, increased abundance of *P. nanceiensis* was associated with greater decline in lung function.	(8, 99, 100)
*Prevotella stercorea*	*Leyella stercorea*	Anaerobic bacterium isolated from human feces and originally described alongside *Prevotella* copri as a novel gut-associated species using phenotypic and 16S rRNA analyses. A later phylogenomic re-evaluation of the genus reassigned the species to Leyella based on genomic distinctiveness from retained Prevotella species.	(7, 8, 17, 90)
*Prevotella intestinalis*	*Palleniella intestinalis*	Gut-associated anaerobic bacterium shown to enhance susceptibility to mucosal inflammation following gut microbiome perturbation. In genetically resistant mice, *P. intestinalis* promoted arthritis through mucosal innate immune activation, while additional studies identified the species in gut microbiome and hepatic transcriptomic analyses associated with hepatocellular carcinoma development in metabolic dysfunction-associated steatohepatitis models.	(17, 101–103)

^*a*^ Because much of the foundational microbiome literature predates recent phylogenomic and taxonomic revisions within *Prevotellaceae*, many studies attributed to *Prevotella* involve species now reassigned to related genera, including *Segatella*, *Leyella*, *Hoylesella*, *Palleniella*, and *Xylanibacter*. This table provides a concise framework for interpreting historical findings within the context of updated taxonomic classifications.

## ECOLOGY OF *PREVOTELLA* TAXA RETAINED AFTER RECLASSIFICATION

**Fig 2:**
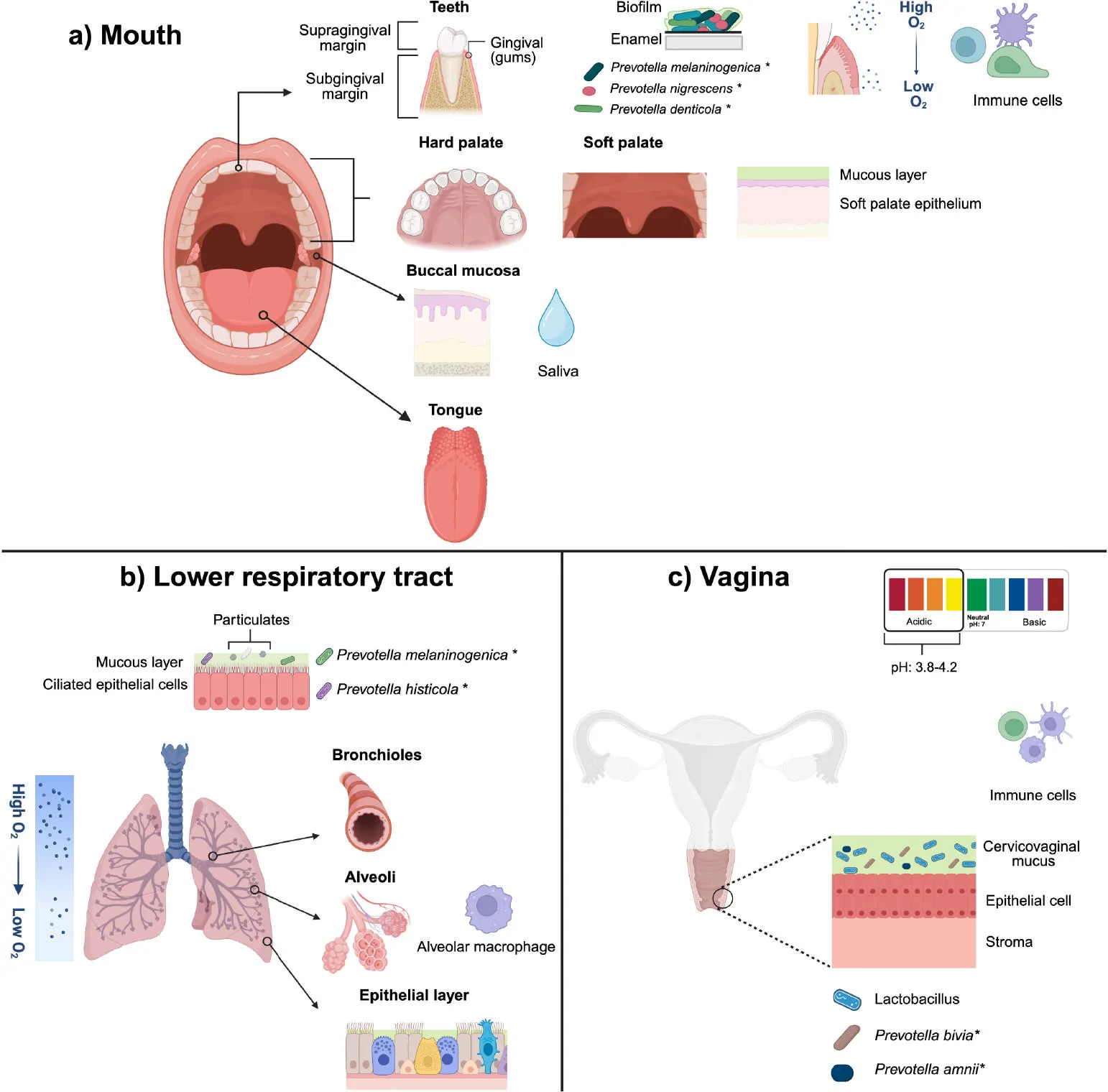
Environmental features of the mouth (**a**), lower respiratory tract (**b**), and vagina (**c**). Ecological and structural differences exist among the areas of the human body, with each region comprising environmental niches with a differing bacterial and structural composition, as well as pH and oxygen levels. Created with https://BioRender.com*.*

Despite our knowledge of *Prevotellaceae* prevalence in the human microbiome based on sequencing data sets, experimentally studying its functional roles has been challenging due to technical and ecological factors. These include difficulties in collecting targeted specimens from sites such as the airways; isolating and maintaining cultures of these fastidious organisms; a lack of protocols and resources for understanding their distinct nutritional requirements and growth characteristics; a shortage of genetic tools for manipulation; limitations of 16S rRNA sequencing in differentiating closely related species in microbiomes; and the higher cost of higher-resolution whole-genome sequencing. Furthermore, microbial composition varies significantly across individual human microbiomes, and each distinct body niche has unique structural, chemical, and microbial features (Fig. 2). Therefore, examining *Prevotellaceae* within discrete anatomical niches is essential for understanding how local environmental pressures shape its persistence and functional activity.

The remaining *Prevotella species* retained after reclassification appear to be unified by their ecology, predominantly colonizing oxygenated human mucosal sites, including the mouth, airways, lungs, and vagina. The recent reclassification of the well-studied human gut species *Prevotella copri* to *Segatella copri* refines our understanding of phylogenetic relationships within the *Prevotellaceae* but also highlights a critical knowledge gap regarding the physiology and function of the remaining *Prevotella* species. Because *Prevotella copri* (now *Segatella copri*) had been the focus of much of the experimental and genomic work previously attributed to the *Prevotella* genus, comparatively little is known about the metabolic capabilities, host interactions, and ecological roles of other *Prevotella* species. This gap is particularly important for mucosal sites, such as the mouth, airways, and lungs, where microbiome studies consistently report high abundances of *Prevotella* spp., yet their contributions to health and disease remain poorly understood.

Consistent with metagenomic evidence of diversity, specific *Prevotellaceae* species show differential prevalence in health and disease. Prasoodanan et al. identified 30 *Prevotellaceae* genomes that differed significantly between inflammatory bowel disease (IBD) patients and healthy controls, with eight enriched in IBD samples, including two unclassified taxa. Notably, *P. intermedia*, a species associated with oral inflammatory conditions and opportunistic infections, was among those enriched in IBD, supporting the idea that certain *Prevotellaceae* species may function as pathobionts under inflammatory conditions. These IBD-associated genomes were also enriched for virulence- and inflammation-related functional traits, particularly in Western populations (104). This reinforces the concept of a “mouth–gut axis,” where oral *Prevotella* strains may transit to and persist in the gut under dysbiotic conditions, potentially contributing to disease pathogenesis.

Although *P. copri* (now *Segatella copri*) and *P. stercorea* (now *Leyella stercorea*) are the most abundant gut-associated members of the formerly defined *Prevotella* genus, numerous species that remain classified within *Prevotella*, such as *Prevotella corporis*, *Prevotella disiens*, and *Prevotella falsenii*, are also detected within the gastrointestinal tract, highlighting the considerable ecological diversity of *Prevotellaceae* in the gut environment (17). Large-scale genomic and metagenomic surveys confirm that fecal microbiomes contain a broad array of *Prevotellaceae*-related taxa across populations. Strain-level analyses by Schmidt et al. further demonstrated extensive oral-to-gut microbial transmission, showing that many salivary taxa, including anaerobes such as *Prevotella*, are also present in paired stool samples from the same individuals (105). Complementary work revealed marked geographic and dietary variation in prevalence, with non-Western, high-fiber populations exhibiting greater richness and abundance of species formerly classified as *Prevotella* (106, 107). Together, these findings indicate that this taxonomic group (including both historically classified *Prevotella* and its reclassified relatives) is diverse, geographically structured, and ecologically dynamic.

The oral cavity is a highly heterogeneous environment comprising the tongue, palates, teeth, and buccal mucosa, each of which supports diverse bacterial communities (108). In the oral cavity, surfaces such as the tongue, soft palate, and buccal mucosa constantly shed and replace their outer epithelial cells. This natural cell turnover helps remove attached bacteria and prevents the long-term buildup of biofilms. In contrast, non-shedding surfaces, such as teeth, do not undergo this cell turnover, allowing bacteria to adhere, accumulate, and form stable biofilms, such as dental plaque. As a result, each surface supports distinct microbial communities, contributing to the unique biochemical and structural characteristics found across different regions of the mouth. This environment is further supported by the presence of nutrient-rich saliva, which maintains a pH range of 6–7.5 (109). In addition to this topographical complexity, the mouth is considered the second most populated microbial environment after the gut (110, 111).

Among oral habitats, the tongue is among the most densely colonized surfaces, harboring dominant genera such as *Rothia*, *Actinomyces*, *Neisseria*, and *Streptococcus* (112). Sequencing studies further demonstrate that *Prevotella melaninogenica* and *Veillonella parvula* are highly prevalent in saliva and along the lateral and dorsal surfaces of the tongue, whereas *Streptococcus mitis* and *S. oralis* are more enriched in soft tissue sites, such as the ventral tongue, hard palate, and vestibular mucosa (113). These spatial distributions reflect not only niche-specific environmental pressures but also cooperative microbial interactions. Oral bacteria exist within structured, spatially organized consortia in which metabolically complementary taxa align in micron-scale architectures that facilitate nutrient exchange and community stability (114).

A central mechanism underlying this organization is mutualistic cross-feeding, the reciprocal exchange of metabolites that supports biofilm homeostasis. Through the sharing of primary metabolites, catabolic by-products, cofactors, and even electron acceptors, metabolically interdependent species enhance collective fitness in nutrient-variable environments. Such syntrophic interactions drive biofilm stratification and emergent properties that cannot be predicted from single-species models alone (115). These cooperative dynamics are foundational to oral biofilm development.

Biofilms themselves are highly structured, polymicrobial communities that vary between individuals and anatomical sites (116). Their formation proceeds through sequential stages of attachment, microcolony formation, maturation, and dispersion (117), and their composition shifts in response to diet, oral hygiene, and host immune status (108). Although most apparent on non-shedding surfaces such as teeth, biofilms also form in subgingival crevices and other protected niches. These environments are characterized by steep oxygen gradients, with subgingival regions being markedly more hypoxic than supragingival sites (117, 118). This may have important implications for community assembly and metabolic flexibility of the microbes that inhabit these regions.

In contrast to the oral cavity, the vaginal microbiome exhibits lower taxonomic diversity. The vaginal microbiome of healthy adult women is typically characterized by the dominance of one or more *Lactobacillus* species (119). These lactic acid–producing bacteria play a critical role in maintaining the acidic pH (3.8–4.4) of the vagina, which inhibits the growth of other bacterial pathogens. The vaginal environment is influenced by a variety of factors, including the menstrual cycle, antibiotic and contraceptive use, sexual activity, hormonal fluctuations, and hygiene practices. For example, hormones such as estrogen are key factors in glycogen production, which *Lactobacillus* species utilize to produce lactic acid (120). Thus, the glycogen-rich vaginal environment provides a consistent reservoir for lactic acid production. Under dysbiotic conditions, however, several *Prevotella* taxa retained after reclassification (including *Prevotella bivia, Prevotella disiens*, *Prevotella amnii*, and *Prevotella corporis*) have been shown to increase in abundance within the vaginal microbiome. These species have been associated with bacterial vaginosis, elevated inflammatory cytokine production, epithelial barrier disruption, and increased susceptibility to sexually transmitted infections (121, 122).

## *PREVOTELLACEAE*: BEYOND THE FRIEND OR FOE DICHOTOMY

Although frequently discussed in the context of inflammation and dysbiosis (123), accumulating evidence suggests that members of *Prevotellaceae* are not uniformly pathogenic. Instead, these organisms likely occupy a functional spectrum ranging from symbionts and metabolic collaborators to context-dependent pathobionts. Members of the *Prevotella* genus, including *P. melaninogenica*, *P. histicola*, and *P. corporis*, are common constituents of healthy oral, respiratory, gastrointestinal, and vaginal microbiomes. In these environments, they participate in essential metabolic processes such as the fermentation of complex carbohydrates and the production of short-chain fatty acids (SCFAs), including propionate and acetate (124, 125). These metabolites can support mucosal integrity, immune homeostasis, and host energy balance, suggesting that retained *Prevotella* species may contribute positively to host health under appropriate ecological conditions (126). Importantly, this functional diversity is also evident within the airway, where different retained *Prevotella* species exhibit distinct immunological effects. For example, *P. melaninogenica* has been shown to attenuate inflammatory responses and protect against airway inflammation, whereas *P. intermedia* does not demonstrate the same protective phenotype, highlighting substantial species-specific functional divergence within *Prevotella* diversity (127).

The impact of *Prevotellaceae* on host physiology is likely highly context-dependent, influenced by ecological and host factors such as diet, immune status, and microbial community structure. Under certain conditions, *Prevotellaceae* species can transition from a commensal to a proinflammatory behavior. For instance, *S. copri* (formerly *P. copri*), a common gut symbiont in non-Western populations, has been associated with both beneficial carbohydrate metabolism and inflammatory phenotypes. It improves glucose metabolism in high-fiber diets but has been linked to elevated systemic inflammation and rheumatoid arthritis in other contexts (128). Similarly, oral *Prevotella* spp., such as *P. intermedia* and *P. nigrescens*, are benign residents of the oral cavity under normal conditions but may contribute to periodontal inflammation when ecological balance is disrupted (56, 129, 130). This variability illustrates that the role of *Prevotella* cannot be universally categorized as beneficial or harmful; instead, it reflects the microbial and immunological context in which these species reside.

Furthermore, the *Prevotella* genus exhibits remarkable functional and genetic diversity, even among closely related strains. This diversity is evident in the strikingly different ecological and immunomodulatory properties they can display. Comparative genomics has revealed extensive heterogeneity in gene content related to carbohydrate metabolism, adhesion, and immune signaling among *Prevotella* lineages (8). This indicates niche-specific adaptations and variable host interactions. Consequently, it is crucial to consider species- and strain-level resolution when interpreting associations with health and disease. In essence, *Prevotella* exemplifies the complexity of mucosal microbial ecology. These taxa can function as beneficial symbionts in one context and as inflammatory opportunists in another, depending on factors such as host diet, immunity, and microbial community composition.

## ASSOCIATIONS ACROSS MUCOSAL SITES AND OPPORTUNITIES FOR FUTURE INVESTIGATION

The emergence of high-throughput microbiome sequencing technology revolutionized our understanding of the richness of human-associated microbes and revealed critical knowledge gaps. This paradox of high abundance and low functional clarity establishes a rich new landscape for further study. Modern multi-omics approaches have revolutionized our view of its distribution. These tools have consistently revealed that *Prevotella* species dominate the oral and airway (among other mucosal sites) microbiota and are strongly associated with healthy and diseases, such as periodontitis, asthma, COPD, and cystic fibrosis. Despite this progress in defining prevalence and association, a vast gap remains in understanding the underlying physiology and niche-specific function of *Prevotella*.

Our research group is actively working to close this functional gap through focused genomic and physiological studies. We recently published the complete genome of the oral species *Prevotella histicola* (46) to complement ongoing mechanistic investigations. In a separate preprinted study, we used a high-throughput approach to define carbon utilization in *Prevotella melaninogenica*, identifying a putative polysaccharide utilization locus (PUL) for starch degradation (131). This mechanistic work is crucial groundwork for understanding *Prevotella* ecological significance; for instance, recent findings by El Hafi et al. demonstrated that *P. melaninogenica* participates in cross-feeding organic acids with the key pathogen *Pseudomonas aeruginosa* in the context of CF-associated biofilms (67). These studies collectively underscore that defining the physiology of *Prevotella* requires moving beyond isolation studies to investigate specific metabolic outputs and polymicrobial partnerships. This focus guides the remainder of this review, which examines specific evidence for the unclear role of *Prevotella* within the distinct ecological and disease contexts of the human oral cavity, airways, and vagina.

### Periodontal disease

The oral cavity is colonized by several *Prevotella* species. A metagenomic study investigating microbial prevalence in the human body identified *Prevotella* as the second-most abundant genus in the oral cavity (7). Many commercially available *Prevotella* species were initially isolated from cases of periodontal disease, dental caries, and other oral infections (132). This is generally attributed to the oral cavity’s diverse environmental niches, which support colonization and proliferation by both symbiotic and pathogenic microbes. Changes in diet, hygiene, medical conditions, and other external factors can disrupt this balance, leading to dysbiosis. Such shifts can alter the microbial composition and microbe–microbe and microbe–host interactions (43, 133, 134). Periodontitis is characterized by increased inflammation of the gingival area and subsequent loss of structural integrity (134). While pathogenic biofilms are a prerequisite for its development, pinpointing the specific role of *Prevotellaceae* in progression is complicated due to species-level differences in biofilm formation; for example, *Prevotella nigrescens* forms more robust biofilms than Hoylesella oralis (formerly *P. oralis*) and *Hoylesella loescheii* (formerly *P. loescheii*) (135). Furthermore, the strong association of species such as *Porphyromonas gingivalis* and other *Bacteroidetes* with periodontitis is complicated by their presence in healthy oral flora, limiting their use as definitive diagnostic markers (136). Therefore, to comprehensively define the role of *Prevotella* and other genera in oral health and disease, it will be essential to investigate *Prevotella* chemical and molecular interactions within biofilms, focusing on how polymicrobial communities behave, communicate, and interact with the host immune system to influence both homeostasis and disease pathogenesis.

### Chronic obstructive pulmonary disease (COPD)

COPD is a collection of lung diseases characterized by irreversible obstruction of airflow to the airways and alveoli (137). Individuals diagnosed with COPD also experience extrapulmonary comorbidities, such as cardiovascular diseases, neurological and skeletal defects, and increased neutrophilic inflammation (138–140). As one of the 10 leading causes of death in the United States (141), research into the microbial populations that contribute to the exacerbation of the disease has identified a decrease in the alpha diversity of microbial communities. Ramsheh and colleagues performed a large 16S rRNA gene sequencing analysis of bronchial brush microbiota in patients with chronic obstructive pulmonary disease (COPD) and healthy age-matched controls. The study included samples from 546 individuals (339 with COPD and 207 healthy) across multiple European centers. They found significant differences in airway bacterial composition between COPD patients and healthy individuals. Notably, *Prevotella* was among the genera with the strongest distinctions: its median relative abundance was significantly higher in healthy subjects than in COPD patients (approximately 47.7% vs 33.5%, respectively; *P* < 0.0001). *Prevotella* abundance was also inversely correlated with disease severity, positively correlated with lung function and exercise capacity, and, in corticosteroid-treated COPD patients, correlated with expression of genes involved in epithelial defense. In contrast, other taxa, such as *Streptococcus* and *Moraxella*, showed different patterns across health and disease groups (142). These results indicate that *Prevotella* is more prevalent in the lower airways of healthy individuals and decreases with COPD progression, challenging interpretations that it is uniformly reduced in health or disease contexts.

Einarsson et al. investigated the microbial community composition of the lower airways in adults with chronic obstructive pulmonary disease (COPD), smokers without airway disease, and healthy individuals using both culture-based methods and high-throughput 16S rRNA gene sequencing. The authors found that the microbial communities in COPD patients differ significantly from those in smokers and healthy controls. Specifically, overall community diversity (α and β diversity) was significantly reduced in the lower airway microbiota of individuals with COPD compared with healthy participants. Members of the phylum *Bacteroidetes*, including *Prevotella* spp., were observed at higher relative abundance in the healthy comparison groups (smokers without disease and healthy non-smokers) than in COPD patients. In contrast, some taxa, such as *Pseudomonas*, demonstrated greater prevalence in COPD individuals, although this appeared to be largely driven by a subset of patients in the cohort (143).

These findings indicate a distinct microbial signature associated with COPD, marked by diminished diversity and differential taxonomic representation compared with non-diseased airways. Using culture-independent sequencing, the study showed that taxa within *Bacteroidetes* (including *Prevotella*) were more abundant in healthy and smoker control groups than in the COPD cohort, supporting the notion that *Prevotella* abundance is associated with respiratory health rather than disease status. Consistent with previous work on airway microbiomes, *Prevotella* and other *Bacteroidetes* taxa have been found at higher relative abundance in the lower airways of healthy individuals compared with patients with chronic obstructive pulmonary disease (COPD), highlighting that shifts in microbial community structure and diversity are linked with disease status rather than a uniform enrichment of *Prevotella* in pathology.

The leading causes of COPD are cigarette smoking, secondhand smoke, and/or prolonged exposure to air pollutants (144). Microbial profiling has shown that, while patients with COPD have lower microbial diversity, core genera such as *Pseudomonas*, *Streptococcus*, *Prevotella*, and *Fusobacterium* are still present in healthy and diseased individuals (145, 146). The presence of *Prevotella* in healthy airway microbiomes suggests a protective role that may help limit inflammation and disease severity. However, further longitudinal studies are required to determine whether specific *Prevotella* species can be harnessed for therapeutic purposes. Integrating metagenomic and transcriptomic data will be essential in identifying species-level interactions and understanding their roles in immune regulation and disease progression.

### Asthma

Asthma is characterized by chronic inflammation or obstruction of the respiratory tubes. It can be triggered by allergens, irritants, exercise, or cold, and its symptoms include difficulty breathing, chest tightness, and frequent respiratory infections (147). Treatment includes rescue inhalers that relax the lung muscles and restore oxygen flow. While the exact cause of asthma remains unknown, technological advances have helped elucidate the microbial populations present at different stages of asthma. A study investigating oropharyngeal microbial communities in children and adolescents with varying severities of asthma identified *Streptococcus*, *Veillonella*, *Haemophilus*, *Prevotella*, and *Rothia* as the most dominant genera (148). Moreover, *Prevotella intermedia*, commonly associated with periodontal disease, has been positively linked to severe asthma (149). However, the specific roles and relative abundances of *Prevotella* species in asthma remain to be elucidated and require further investigation.

### Cystic fibrosis

Cystic fibrosis (CF) is a genetic disorder that affects the cystic fibrosis transmembrane conductance regulator (CFTR) gene. This transmembrane ion channel facilitates water-electrolyte movement in various tissues, including the lower and upper airways (150). This highly heterogeneous disease has up to 1,085 CF-causing variants and 55 variants of varying clinical consequences (151). The clinical manifestation of a dysfunctional CFTR gene in CF is caused by abnormal antioxidant composition on the airway surface liquid of the conducting airways. This can worsen inflammation in the lungs’ epithelial tissue, creating an opportunity for bacterial infection, such as *Pseudomonas aeruginosa*, *Haemophilus influenzae*, and *Staphylococcus aureus* (152–154). Anaerobic species such as *Segatella salivae* (previously *Prevotella salivae*) and *P. melaninogenica* have been persistently found in patients’ CF sputum samples (155). These species may originate from the oropharynx and contribute to lower airway colonization and tissue damage. Though the exact pathogenic role of *Prevotella* in CF is unclear, some studies suggest that they may support the growth of other pathogenic bacteria, such as members of the *Streptococcus anginosus* groups (156). While the genomic diversity of CFTR mutations is well documented, their specific effects on tissue structure and susceptibility to microbial colonization remain underexplored.

Multiple studies have demonstrated that *Prevotellaceae* are prevalent and frequently abundant members of the cystic fibrosis (CF) airway microbiome (157–159). These species often coexist with well-known CF pathogens, such as *Pseudomonas aeruginosa* and *Staphylococcus aureus*. Next-generation sequencing has revealed diverse anaerobic communities in sputum and lower airways. In children and adults with CF, *Prevotellaceae* are frequently among the most prevalent genera detected, indicating a complex polymicrobial ecosystem rather than single-pathogen dominance (159, 160). *Prevotella* spp. and other obligate anaerobes are frequently found in high relative abundance in CF sputum and lavage samples, sometimes at levels comparable to *Pseudomonas species*, particularly in early or stable phases of disease (161, 162). Certain taxa such as *Hoylesella nanceiensis* (previously *Prevotella nanceiensis*) and *Porphyromonas pasteri* have been associated with accelerated lung function decline and inflammatory signatures in CF airways, suggesting that anaerobes may contribute to the overall disease process or serve as biomarkers of microbial community shifts (99).

*In vitro* studies have demonstrated that certain *Prevotella* strains possess the ability to inhibit biofilm formation by *Pseudomonas aeruginosa*. For instance, supernatants conditioned by *P. intermedia* and *P. nigrescens* exhibited a significant reduction in the total biofilm biomass of P. aeruginosa clinical isolates, reaching up to approximately 90%. Remarkably, this reduction did not suppress the planktonic growth of *P. aeruginosa*, suggesting an antagonistic interaction that impairs biofilm development (163). This is particularly important as biofilm formation is a crucial virulence factor in chronic obstructive pulmonary disease (COPD) lung infections. Other *Prevotella* spp., such as *P. histicola* and *P. nigrescens*, have been reported to dampen pro-inflammatory responses induced by *P. aeruginosa* in airway epithelial cells, reducing TLR-4 and NF-κB signaling and lowering IL-6/IL-8 release, which could modulate inflammation in polymicrobial infections (164).

Emerging research indicates that *P. aeruginosa* may facilitate the survival of certain *Prevotella* spp. within polymicrobial communities through metabolic cross-feeding. A cystic fibrosis-relevant model demonstrated that *P. aeruginosa* supports the survival of *P. melaninogenica*, which cannot survive independently in the model, likely by providing metabolites such as short-chain fatty acids or other nutrients (67). This type of metabolic interaction highlights the possibility that, contrary to their competitive nature, *Pseudomonas* and *Prevotella* can establish mutually beneficial or commensal relationships under specific conditions. This dynamic contributes to the complexity of community interactions and influences the persistence dynamics of these microorganisms.

Community network analyses indicate that *P. aeruginosa* often shows negative correlations with anaerobes such as *Prevotella* in CF microbiome data sets, suggesting potential competitive interactions under particular contexts or stages of disease progression (165). Competition may also be reflected in biofilm spatial organization and nutrient resource partitioning, where *P. aeruginosa* dominance can suppress other taxa while crosstalk occurs in mixed communities.

Although fewer studies focus specifically on *Prevotella-Staphylococcus aureus* interactions, broader ecological reviews in CF indicate that *S. aureus* co-infection dynamics are shaped by polymicrobial networks in which anaerobes, including *Prevotella*, are embedded (157, 166–169). These mixed communities can influence virulence, antibiotic tolerance, and host immune responses and have been linked epidemiologically to variations in disease severity. Understanding the microbe–microbe interactions within CF lungs could provide critical insights into the polymicrobial nature of disease exacerbation and inform the development of targeted therapeutics.

### Bacterial vaginosis

The vaginal microbiota is influenced by genetics, hormones, contraceptives, hygiene practices, sexual intercourse, and the use of antibiotics or probiotics (170). Disruption of this microbial balance by the aforementioned factors can cause dysbiosis, leading to bacterial vaginosis (BV), characterized by watery discharge, a fishy odor, and an elevated pH (>4.5) (171).

BV is widely regarded as a polymicrobial condition, involving a decrease in *Lactobacillus* species and an increase in organisms, such as *Prevotella*, *Gardnerella* spp., *Porphyromonas*, and *Mycoplasma hominis* (172). Although the precise role of *Prevotella* is not fully understood, it is thought to be a key component of this shift. *Gardnerella* is believed to act as an early colonizer, forming biofilms that enable the growth and immune evasion of facultative and strict anaerobes, such as *P. bivia* (173, 174). The acidic pH of a healthy vagina (3.8–4.4) typically inhibits such pathogens, which is maintained by the production of lactic acid from *Lactobacillus* species (171, 175, 176). Furthermore, the combination of specific *Prevotella* species, in tandem with *Gardnerella* species, may increase the likelihood of recurring BV (177).

Standard antibiotic treatment for BV often yields only moderate initial cure rates (~60–70%), with many patients experiencing relapse within months. A key mechanism underlying this treatment failure is the persistence of structured multi-species biofilms on the vaginal mucosa that are refractory to antibiotic eradication. In a clinical study, dense *Gardnerella* spp. and *Fannyhessea vaginae* (formerly *Atopobium vaginae*) biofilms remained adherent to the epithelium after oral metronidazole therapy, providing a reservoir for rapid resurgence of BV-associated bacteria and contributing to recurrence (178). Muzny et al. (179) present an updated conceptual model of BV pathogenesis that integrates recent ecological and mechanistic data on how key BV-associated taxa interact to establish and sustain dysbiosis. This framework moves beyond single-pathogen hypotheses to emphasize a polymicrobial consortium model centered on interactions among *Gardnerella* spp., *Prevotella bivia*, and other anaerobes, such as *Fannyhessea vaginae*, in the formation and maintenance of the BV biofilm. The updated BV model incorporates mutualistic nutrient exchange between *Gardnerella* and *P. bivia*, wherein *Gardnerella* provides amino acids and peptides that support the growth of *P. bivia*, and *P. bivia* supplies ammonia that can be used by *Gardnerella* spp*.,* establishing a cooperative metabolic relationship that enhances colonization and biofilm stability (179).

Pybus et al. investigated metabolic interactions between *Prevotella bivia* and *Gardnerella* in the context of BV. In a defined vaginal medium, they found that free amino acids stimulated *P. bivia* growth, while peptides enhanced the growth of both species. During culture, *P. bivia* produced net ammonia, whereas *Gardnerella* consumed it; moreover, *P. bivia* supernatants enriched in ammonia promoted *Gardnerella* growth (21). These findings provide direct evidence of reciprocal metabolic exchange, in which *P. bivia* supplies ammonia and *Gardnerella* utilizes it, supporting a symbiotic relationship that sustains the polymicrobial community characteristic of BV. This finding underscores that targeting the biofilm lifestyle, rather than planktonic organisms alone, may be necessary to improve treatment efficacy and long-term effectiveness. In addition, this suggests that broad-spectrum antibiotics used to treat the bacterial load are not a reliable long-term treatment plan for BV (177).

Interestingly, Pelayo et al. (180) identified *Prevotella* species, particularly *Hoylesella timonensis* (previously *P. timonensis*), as major contributors of sialidase activity in the vaginal microbiome. Using comparative genomics and multi-omics analyses, the study showed that sialidase genes and transcripts are widespread and highly expressed in *Prevotella*, often exceeding those from *Gardnerella*, previously considered the main sialidase source in BV. These enzymes cleave sialic acids from mucins, weakening the cervicovaginal mucus barrier and degrading IgA, thereby facilitating bacterial adhesion, biofilm formation, and mucosal vulnerability characteristic of BV (180). This finding is important because it broadens our understanding of the enzymatic contributors to BV pathogenesis, highlighting *Prevotella* as a key driver of mucosal barrier disruption and microbial persistence. As a result of advances in clinical diagnostics and sequencing technology, researchers can monitor shifts in the vagina microbiome more precisely. However, future studies should investigate the host’s immune response to persistent and recurring infections, which will be crucial for developing preventive and therapeutic strategies.

In addition to its high prevalence and association with dysbiotic vaginal microbiota profiles, BV has important obstetric implications. Women affected by BV are at increased risk for adverse pregnancy outcomes, most notably preterm birth. Koumans et al. (181) documented the substantial burden of BV among U.S. women of reproductive age, emphasizing that it is often asymptomatic yet highly prevalent (181). Building on this, numerous epidemiological and cohort studies have demonstrated that BV is associated with a 1.4- to 1.8-fold increased risk of preterm delivery, likely mediated through ascending infection and inflammation that can precipitate premature rupture of membranes and uterine contractions (182). In addition to host, behavioral, and environmental factors, sexually transmitted infections (STIs) are important determinants of vaginal microbiota composition and stability. Longitudinal data show that BV and non-ulcerative STIs, such as gonorrhea and chlamydial infection, are reciprocally associated, with BV preceding STI acquisition and STI infection predicting subsequent BV, independent of other risk factors (183). These findings reinforce that STI exposure and co-infection contribute to shifts in vaginal microbial communities, further highlighting the interconnectedness of microbial dynamics and sexual health outcomes. Collectively, these findings highlight that BV is not only associated with unpleasant urogenital symptoms but also contributes to clinically significant reproductive outcomes, reinforcing the need for improved diagnostic and therapeutic strategies to mitigate risks during pregnancy.

To contextualize the role of *Prevotella* within the vaginal microbiome, it is important to consider the community state type (CST) framework originally described by Ravel et al., which grouped vaginal microbiota into five major categories based on dominant taxa (118). In this model, *Lactobacillus*-dominant CSTs (*L. crispatus*, *L. iners*) are generally associated with vaginal health, whereas CST IV is defined by depletion of *Lactobacillus* and enrichment of diverse anaerobes, including *Prevotella*, *Gardnerella*, and *Atopobium*, and is strongly associated with bacterial vaginosis (BV) (184–186).

More recently, this framework has been refined through the development of VALENCIA (VAginaL community state typE Nearest CentroId clAssifier), a nearest-centroid–based approach trained on over 13,000 vaginal microbiome profiles. This updated classification system standardized CST assignment across studies and expanded the number and resolution of defined CST subtypes, particularly within the heterogeneous CST IV category. Rather than representing a single dysbiotic state, CST IV is now recognized as a spectrum of anaerobe-dominant subtypes with distinct compositional and demographic associations. Several of these subtypes are characterized by increased relative abundance of *Prevotella* species, reinforcing their association with *Lactobacillus*-depleted communities and BV (187). The refinement of CST classification, therefore, underscores both the ecological diversity of vaginal anaerobic communities and the recurrent enrichment of *Prevotella* within dysbiotic states.

### Endometriosis

Building on these associations within the vaginal microbiome, recent research has shown that *Prevotella* are also elevated in women with chronic pelvic pain and endometriosis, indicating that their role may extend beyond local dysbiosis to systemic or inflammatory pelvic conditions. In a pilot study, researchers profiled the vaginal and rectal microbiomes of women with chronic pelvic pain (CPP) with endometriosis (CPP-Endo), CPP without endometriosis, and controls without pain or endometriosis using 16S rRNA gene sequencing. They also measured cervicovaginal immune mediators to explore links between microbial profiles and inflammation. Significant differences in microbial composition were observed between groups, suggesting that local microbiome alterations may be associated with CPP and its underlying causes, including endometriosis (188). Consistent with emerging evidence linking the genital microbiome to gynecologic pain syndromes, women with chronic pelvic pain and endometriosis exhibit distinct vaginal microbial profiles characterized by increased abundance of *Prevotella* compared with asymptomatic controls. This enrichment of *Prevotella* in CPP-associated endometriosis parallels findings from other pelvic pain and dysbiotic contexts and supports the notion that specific anaerobic taxa may be involved in the pathophysiology or persistence of endometriosis-related symptoms.

## FUTURE DIRECTIONS IN *PREVOTELLA* BIOLOGY

The newly redefined genus *Prevotella* represents a taxonomically diverse and ecologically complex group of organisms. Analysis of *Prevotellaceae* prevalence and diversity across human niches, including the oral cavity, respiratory tract, and vagina, reveals both their dynamic distribution and potential for functional specialization. While many species seem to be stable symbionts, they are also associated with disease across many conditions, although the evidence does not yet support a direct causal or pathogenic role in disease.

This functional duality suggests that *Prevotella* may act as an ecological barometer in human health. This concept will guide our group’s future study of the genus: the state of *Prevotella* (specifically its expression of virulence factors or its role in biofilm architecture) is not an intrinsic characteristic of the organism that can define host outcomes but rather a direct and potentially measurable indicator of local ecosystem instability caused by inflammation or co-colonization.

**Fig 3:**
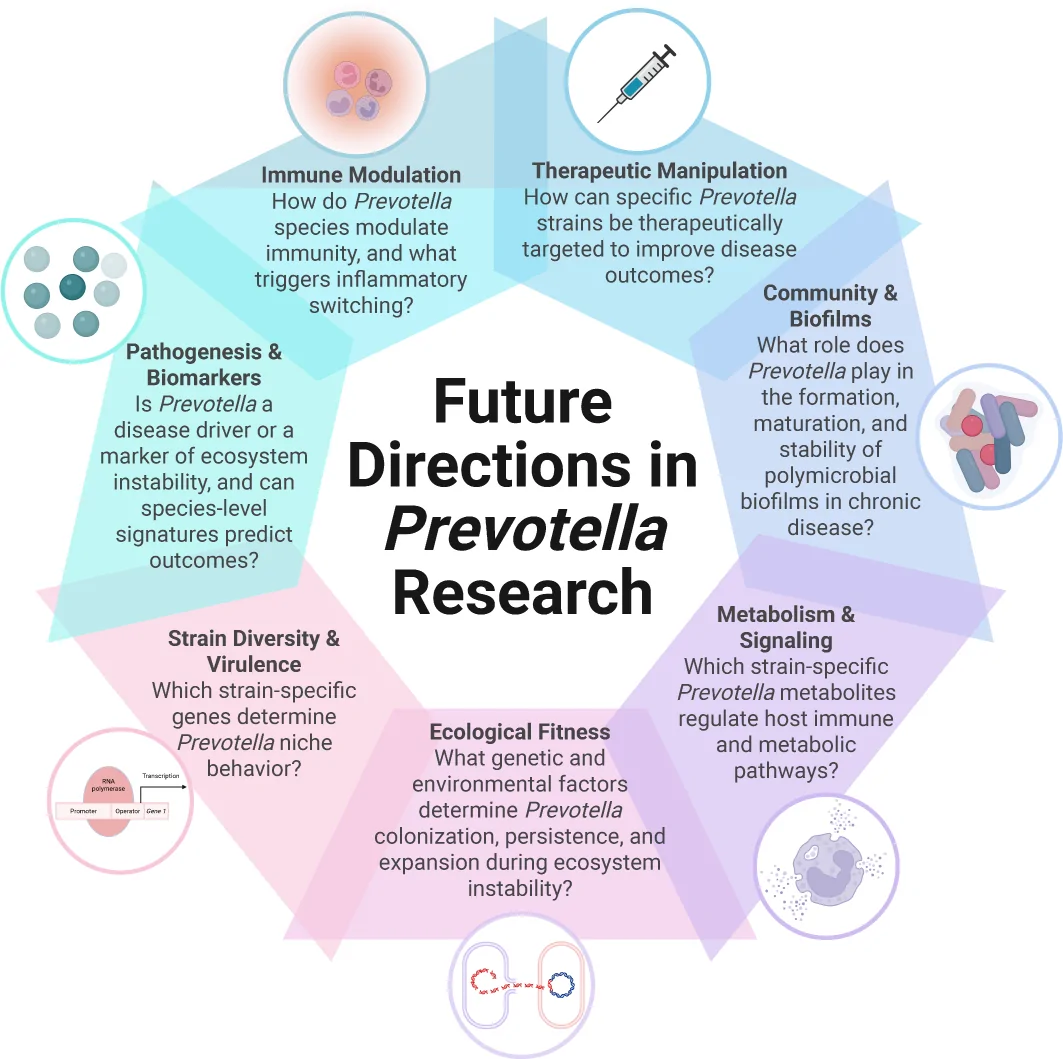
Circular framework highlighting seven priority areas that address major unresolved questions in *Prevotella* biology. Each segment represents a key research domain: immune modulation, therapeutic manipulation, community and biofilms, metabolism and signaling, ecological fitness, strain diversity and virulence, and pathogenesis and biomarkers, paired with a central research question designed to move the field beyond association-based studies. Created with https://BioRender.com.

To test this, research must pivot from a reductionist approach to one that embraces the investigation of microbial context. The mere presence of a *Prevotella* species in a niche is insufficient for determining risk; rather, its role is most likely determined by its location, neighbors, and host immune status. For example, *P. intermedia* may function as a harmless symbiont in the saliva but become a key pathogenic component when integrated into a subgingival biofilm (189, 190). Therefore, we must resist the assumption of functional uniformity and acknowledge that the diverse species distribution across the oral cavity, airways, and vagina necessitates niche-specific investigation to truly clarify the role of these organisms. To move beyond association-based linkages, future research must prioritize species-level genomics and functional studies. To synthesize current knowledge gaps and define a forward-looking research agenda, we propose a conceptual framework outlining seven priority areas for future investigation. These domains span strain diversity, immune modulation, community dynamics, metabolic activity, ecological fitness, disease causality, and therapeutic targeting. By centering each domain around a focused research question, this framework highlights the need to transition from descriptive association studies toward mechanistic, strain-resolved, and translational approaches in *Prevotella* research (Fig. 3). Integrating whole-genome sequencing to identify strain-specific microbes, combined with culture-based morphological and biochemical assays, is essential to clarify the roles that individual *Prevotella* species play in polymicrobial communities, immune regulation, and metabolite production. New experimental tools that allow for the controlled study of polymicrobial communities, host-microbe interactions, and the local immune response will be necessary to understand how these interactions influence health outcomes and ultimately may help us to harness these relationships to develop next-generation therapeutics. This review has traced the evolution of *Prevotella* research from the historical challenges of cultivation to the most recent genomic reclassification, discussing the unclear role of *Prevotella* in health and disease despite high prevalence across human mucosal sites. Recognizing *Prevotella* as a dynamic, context-sensitive member of polymicrobial communities and advancing a deeper mechanistic understanding of its niche-specific functions may ultimately inform targeted strategies to modulate microbial ecosystems in health and disease.

## SUPPLEMENTAL MATERIAL

Table S1 jb.00133-26-s0001.docx Table of previously classified *Prevotella* species, new names, and biological implications.

ASM does not own the copyrights to Supplemental Material that may be linked to, or accessed through, an article. The authors have granted ASM a non-exclusive, world-wide license to publish the Supplemental Material files. Please contact the corresponding author directly for reuse.
