# Supplementary material for: The redefined identity of Prevotella: new implications for oral, respiratory, and vaginal health
Source: J Bacteriol. 2026 Jul 10;208(8):e00133-26. doi: 10.1128/jb.00133-26 (PMC13492131; doi:10.1128/jb.00133-26)
Supplement: Supplementary file 1 [file jb.00133-26-s0001.docx]

| ***Historical Nomenclature*** | ***Current Taxonomy*** | **Reported Biological Attributes** | **References** |
| --- | --- | --- | --- |
| *Prevotella amnii* | *Prevotella amnii* | Vaginal anaerobic species associated with bacterial vaginosis and adverse reproductive health states; detected in cervicovaginal microbial communities linked to inflammation and altered vaginal microbiome composition; Noted as having one of the smallest genomes | ^1–3^ |
| *Prevotella aurantiaca* | *Prevotella aurantiaca* | Oral anaerobic species associated with polymicrobial biofilm communities and periodontitis; identified in subgingival plaque and shown to possess proteolytic activity and inflammatory potential within oral disease-associated microbial communities. | ^4,5^ |
| *Prevotella bivia* | *Prevotella bivia* | Common vaginal species; associated with BV-related ecology and human infections; co-inoculation with Gardnerella vaginalis reproduced BV-like features in mice; recruits CCR5+ TH cells; linked to increased HIV susceptibility. | ^1,2,6–9^ |
| *Prevotella brunnea* | *Prevotella brunnea* | Anaerobic, pigmented oral bacterium identified within human subgingival and oral microbial communities; associated with periodontitis-associated biofilms and characterized as part of the diverse oral anaerobic microbiota. | ^2,10^ |
| *Prevotella communis* | *Prevotella communis* | Strictly anaerobic saccharolytic bacterium isolated from sheep rumen samples in Slovenia and Japan; specialized in degradation of xylan and pectin-related polysaccharides but unable to utilize starch. Also detected in cattle and sheep rumen metagenomes from Scotland and New Zealand. | ^2,11^ |
| *Prevotella corporis* | *Prevotella corporis* | Anaerobic Gram-negative bacterium originally isolated from the human oral cavity and associated with periodontal disease and oral polymicrobial biofilms; more recent studies identified the species within gut and vaginal-associated microbial communities linked to host inflammatory environments. | ^1,2,6,12–15^ |
| *Prevotella dentalis* | *Prevotella dentalis* | Anaerobic Gram-negative bacterium isolated from human oral and dental infections, including periodontal and endodontic sites; associated with oral polymicrobial biofilms and characterized through phenotypic and genomic analyses as a distinct oral Prevotella species. | ^1,2,6,16–18^ |
| *Prevotella dentasini* | *Prevotella dentasini* | Anaerobic bacterium isolated from human dental plaque and oral clinical specimens; associated with periodontal disease-associated microbial communities and further characterized through genomic analyses examining oral Prevotella diversity and taxonomy. | ^2,19,20^ |
| *Prevotella denticola* | *Prevotella denticola* | Anaerobic oral bacterium isolated from human dental and periodontal sites; identified within polymicrobial oral biofilms and characterized through genomic and phylogenetic analyses examining oral Prevotella diversity and taxonomy. | ^1,2,6,21–23^ |
| *Prevotella disiens* | *Prevotella disiens* | Anaerobic bacterium identified in human vaginal and urogenital microbial communities, particularly in association with bacterial vaginosis and dysbiosis; studies also reported its presence in polymicrobial infections and linked the species to inflammatory vaginal environments and altered microbial community structure. | ^1,24,25^ |
| *Prevotella falsenii* | *Prevotella falsenii* | Originally isolated from monkey dental plaque and described as a Prevotella intermedia-like anaerobic species based on phenotypic and 16S rRNA analyses. Later studies identified P. falsenii in gut microbiome datasets associated with DSS-induced colitis and in fecal microbiota analyses examining functional constipation, depression, and anxiety following fecal microbiota transplantation. | ^2,26–28^ |
| *Prevotella fusca* | *Prevotella fusca* | Anaerobic species isolated from the human oral cavity and differentiated from closely related oral Prevotella species using phenotypic and 16S rRNA analyses. Comparative pan-genome analysis later identified P. fusca among oral Prevotella species associated with periodontitis and characterized its genomic repertoire related to virulence and adaptation within periodontal niches. | ^2,29,30^ |
| *Prevotella herbatica* | *Prevotella herbatica* | Anaerobic bacterium isolated from a methanogenic reactor treating cattle farm waste; characterized as capable of decomposing plant polysaccharides, including xylan and pectin-derived substrates, and producing acetate and succinate as major fermentation products. | ^2,31^ |
| *Prevotella histicola* | *Prevotella histicola* | Anaerobic Gram-negative bacterium originally isolated from the human gastrointestinal tract. Studies reported that oral administration of P. histicola modulated immune responses in multiple sclerosis and arthritis models, including expansion of Allobaculum and increased butyrate production. In mice, P. histicola reduced LPS-induced olfactory bulb inflammation associated with rhinitis. In cystic fibrosis bronchial epithelial cells, the species activated alternative NF-κB signaling pathways distinct from those induced by Pseudomonas aeruginosa. A complete genome sequence for strain T05-04 was also reported. | ^1,2,6,32–36^ |
| *Prevotella ihumii* | *Prevotella ihumii* | Anaerobic bacterium isolated from human stool specimens and initially described as a novel species from a healthy woman. Subsequent studies identified P. ihumii in gut microbiome analyses associated with cirrhosis and impaired inhibitory control, while genomic characterization studies examined Cas12a nuclease diversity among closely related bacterial orthologs including P. ihumii. | ^2,37–40^ |
| *Prevotella illustrans* | *Prevotella illustrans* | Anaerobic Gram-negative bacterium isolated from human oropharyngeal abscess puncture fluid; characterized using phenotypic, biochemical, and genomic analyses and proposed as a novel species within the genus Prevotella. | ^2,41^ |
| *Prevotella intermedia* | *Prevotella intermedia* | Anaerobic oral pathogen frequently detected in periodontal disease, odontogenic infections, and extraoral abscesses. Quantitative molecular studies differentiated P. intermedia from P. nigrescens in clinical specimens and demonstrated clonal diversity among isolates from diseased and healthy periodontal sites. A recent review further summarized its involvement in periodontal disease and systemic inflammatory conditions, highlighting virulence-associated traits including biofilm formation, proteolytic activity, and host inflammatory modulation. | ^1,2,6,28,42–45^ |
| *Prevotella jejuni* | *Prevotella jejuni* | Anaerobic Gram-negative bacterium originally isolated from the jejunum of a child with coeliac disease and characterized as a novel Prevotella species using phenotypic and 16S rRNA analyses. A later metagenome-genome-wide association study detected P. jejuni within oral microbiome datasets and identified associations between oral microbial composition and host genetic loci influencing salivary and tongue dorsum microbiota profiles. | ^2,46,47^ |
| *Prevotella koreensis* | *Prevotella koreensis* | Anaerobic Gram-negative bacterium isolated from human subgingival dental plaque collected from a periodontitis lesion; characterized using phenotypic, biochemical, chemotaxonomic, and genomic analyses and proposed as a novel species within the genus Prevotella. | ^2,48^ |
| *Prevotella lacticifex* | *Prevotella lacticifex* | Anaerobic ruminal bacterium characterized as a lactate-producing member of the rumen microbiota with carbohydrate fermentation capabilities. A later structural equation modeling study identified associations between rumen bacterial abundance, including P. lacticifex, and lactating dairy cow host phenotypes related to production and physiological traits. | ^2,49,50^ |
| *Prevotella marseillensis* | *Prevotella marseillensis* | Anaerobic Gram-negative bacterium isolated from the stool of a patient with recurrent Clostridium difficile infection | ^2,51^ |
| *Prevotella melaninogenica* | *Prevotella melaninogenica* | Pigmented anaerobic Gram-negative bacterium associated with oral and respiratory environments. Recent studies characterized its starch utilization locus and carbohydrate metabolism using a defined basal medium assay system. In cystic fibrosis lung polymicrobial communities, P. aeruginosa supported P. melaninogenica survival through metabolic cross-feeding interactions. Another study reported enrichment of P. melaninogenica in the lower respiratory tract of patients with checkpoint inhibitor pneumonitis and radiation pneumonitis. | ^1,2,6,52–54^ |
| *Prevotella merdae* | *Prevotella merdae* | Anaerobic Gram-negative bacterium isolated from human feces and characterized as a novel species using phenotypic, biochemical, and genomic analyses. A later clinical study reported associations between gut microbiota composition, including P. merdae, and improved responses to anti-PD-1 immunotherapy following fecal microbiota transplantation in patients with refractory metastatic solid cancers. | ^2,55,56^ |
| *Prevotella micans* | *Prevotella micans* | Anaerobic Gram-negative bacterium isolated from the human oral cavity and described as a novel species using phenotypic and 16S rRNA analyses. A later study investigating tryptophanase-mediated indole production in Prevotella identified molecular mechanisms contributing to indole biosynthesis among oral Prevotella species including P. micans. | ^1,2,57,58^ |
| *Prevotella multiformis* | *Prevotella multiformis* | Anaerobic Gram-negative bacterium isolated from human subgingival plaque | ^2,59^ |
| *Prevotella nigrescens* | *Prevotella nigrescens* | Anaerobic oral bacterium associated with periodontal disease, odontogenic infections, and extraoral abscesses. Molecular studies differentiated P. nigrescens from P. intermedia in clinical specimens and demonstrated clonal variation among isolates recovered from healthy and diseased periodontal sites. | ^1,2,6,13,42–44^ |
| *Prevotella pallens* | *Prevotella pallens* | Pigmented anaerobic bacterium proposed as a novel species isolated from the human oral cavity and differentiated from related oral Prevotella species through phylogenetic and phenotypic analyses. Subsequent studies examined β-lactamase production among P. pallens genotypes and evaluated in vitro susceptibility to antimicrobial agents commonly used against anaerobic oral pathogens. | ^1,2,6,44,60^ |
| *Prevotella pectinovora* | *Prevotella pectinovora* | Anaerobic bacterium characterized as a novel pectin-degrading species based on phenotypic and genomic analyses; capable of utilizing pectin and other plant-derived polysaccharides, supporting a role in carbohydrate degradation. | ^1,2,61^ |
| *Prevotella phocaeensis* | *Prevotella phocaeensis* | Anaerobic Gram-negative rod isolated from the stool of a patient with Clostridium difficile infection during a culturomics study of the human gut microbiota. The species was characterized using phenotypic testing, MALDI-TOF MS, and noncontiguous finished genome sequencing and proposed as a novel species within the genus Prevotella. | ^2,6,62^ |
| *Prevotella rectalis* | *Prevotella rectalis* | Strictly anaerobic Gram-negative rod isolated from a human rectal sample obtained during a culturomics study. The species was characterized by MALDI-TOF MS, phenotypic and biochemical testing, fatty acid analysis, and whole-genome sequencing, which supported its designation as a novel species within the genus Prevotella. | ^2,63^ |
| *Prevotella scopos* | *Prevotella scopos* | Anaerobic bacterium isolated from the human oral cavity and described as a novel species using phenotypic characterization and 16S rRNA gene analyses alongside Prevotella fusca. | ^1,2,29^ |
| *Prevotella veroralis* | *Prevotella veroralis* | Anaerobic oral bacterium identified as the causative organism in a rare lung abscess case diagnosed using third-generation metagenomic sequencing. | ^1,2,23^ |
| *Prevotella vespertina* | *Prevotella vespertina* | Anaerobic Gram-negative bacterium isolated from a human abscess in a hospitalized patient and characterized as a novel species using phenotypic, biochemical, MALDI-TOF MS, and genomic analyses. | ^1,2,64^ |
| *Prevotella tannerae* | Alloprevotella tannerae | Anaerobic oral bacterium isolated from the human gingival crevice and described alongside Prevotella enoeca using phenotypic, biochemical, and fatty acid analyses. The species was distinguished from related oral Prevotella taxa by its saccharolytic profile and enzyme activity patterns. A later phylogenomic re-evaluation recognized the species as belonging to Alloprevotella rather than retained Prevotella. | ^6,65^ |
| *Prevotella bergensis* | *Hallella bergensis* | Skin-specific gene repertoire enriched for signal transduction, carbohydrate metabolism, and inorganic ion metabolism genes.Anaerobic Gram-negative rod isolated from human clinical specimens, including skin and soft tissue infections; characterized as pigmented, saccharolytic, and phylogenetically distinct from closely related Prevotella species based on 16S rRNA gene analysis. | ^1,66,67^  . |
| *Prevotella colorans* | *Hallella colorans* | Anaerobic bacterium isolated from the human gastrointestinal tract and human wound specimens; identified within host-associated anaerobic microbial communities and later reclassified within the genus Hallella. | ^2,68,69^ |
| *Prevotella buccalis* | *Hoylesella buccalis* | Vaginal/gut-associated species with higher prevalence in seniors in Westernized populations; Macrolide resistance gene *ermF* prevelance | ^1,2,70^ |
| *Prevotella enoeca* | *Hoylesella enoeca* | Anaerobic bacterium isolated from the human gingival crevice and described as a novel oral species alongside Prevotella tannerae using phenotypic, biochemical, and fatty acid analyses. The species was differentiated from related oral Prevotella taxa by its carbohydrate utilization and enzyme activity profiles. A later phylogenomic re-evaluation reassigned the species to the genus Hoylesella. | ^6,65^ |
| *Prevotella loescheii* | *Hoylesella loescheii* | Anaerobic bacterium reported as the causative agent of a bacteremic skin and soft tissue infection in a human patient, highlighting its potential role in invasive extraoral disease. A later phylogenomic analysis of the genus reassigned P. loescheii to the newly established genus Hoylesella. | ^1,2,6,71^ |
| *Prevotella marshii* | *Hoylesella marshii* | Anaerobic bacterium originally isolated from the human oral cavity and described as a novel species based on phenotypic and 16S rRNA analyses. A later clinical case report identified H. marshii as the causative organism in a pleural infection. Subsequent phylogenomic analysis reassigned the species from Prevotella to the genus Hoylesella. | ^6,72,73^ |
| *Prevotella oralis* | *Hoylesella oralis* | Originally described as Bacteroides oralis, an anaerobic bacterium isolated from the human oral cavity. The species was later reassigned to the genus Prevotella during broader taxonomic restructuring of pigmented anaerobic oral bacteria. Subsequent phylogenomic analysis further reassigned the species to the genus Hoylesella based on genomic and phylogenetic distinctiveness from retained Prevotella species. | ^1,2,6,74,75^ |
| *Prevotella shahii* | *Hoylesella shahii* | Anaerobic bacterium isolated from the human oral cavity and described as a novel species using phenotypic and 16S rRNA analyses alongside Prevotella salivae. A later phylogenomic re-evaluation of the genus reassigned the species to Hoylesella. | ^6,76^ |
| *Prevotella timonensis* | *Hoylesella timonensis* | Anaerobic bacterium originally isolated from a human breast abscess and described as a novel species using phenotypic and molecular analyses. Later studies demonstrated that P. timonensis degrades the vaginal epithelial glycocalyx through high fucosidase and sialidase activity and enhances HIV-1 uptake in vaginal models by differentially affecting primary dendritic cell subsets. A subsequent phylogenomic re-evaluation reassigned the species to the genus Hoylesella. | ^1,6,77–79^ |
| *Prevotella nanceiensis* | *Hoylessa nanceiensis* | Anaerobic bacterium originally isolated from human clinical specimens and characterized as a novel Prevotella species using phenotypic and molecular analyses. A later phylogenomic study reassigned the species to the genus Hoylesella. In cystic fibrosis sputum microbiota, increased abundance of P. nanceiensis was associated with greater decline in lung function. | ^6,80,81^ |
| *Prevotella lascolaii* | *Leyella lascolaii* | Anaerobic Gram-negative bacterium isolated from the vaginal flora of a patient with bacterial vaginosis during a microbial culturomics study; characterized using phenotypic, MALDI-TOF MS, and genomic analyses and proposed as a novel species within the genus Prevotella. A later phylogenomic re-evaluation of the genus reassigned the species to the newly established genus Leyella. | ^6,82^ |
| *Prevotella stercorea* | *Leyella stercorea* | Anaerobic bacterium isolated from human feces and originally described alongside Prevotella copri as a novel gut-associated species using phenotypic and 16S rRNA analyses. A later phylogenomic re-evaluation of the genus reassigned the species to Leyella based on genomic distinctiveness from retained Prevotella species. | ^1,2,6,83^ |
| *Prevotella intestinalis* | *Palleniella intestinalis* | Gut-associated anaerobic bacterium shown to enhance susceptibility to mucosal inflammation following gut microbiome perturbation. In genetically resistant mice, P. intestinalis promoted arthritis through mucosal innate immune activation, while additional studies identified the species in gut microbiome and hepatic transcriptomic analyses associated with hepatocellular carcinoma development in metabolic dysfunction-associated steatohepatitis models. | ^2,84–86^ |
| *Prevotella albensis* | *Segatella albensis* | One of the best-known non-human-host members; Anaerobic ruminal bacterium isolated from sheep rumen; capable of fermenting a variety of carbohydrates and producing succinate and acetate as major metabolic end products, suggesting a role in plant polysaccharide degradation within the rumen environment. | ^1,2,87,88^ |
| *Prevotella asaccharophila* | *Segatella asaccharophila* | Obligate anaerobic, Gram-negative, non-motile pectinophilic bacterium isolated from a two-phase methane fermentation system; preferentially utilized pectin, d-galacturonate, and d-glucuronate rather than common sugars such as glucose, and produced succinate and acetate as major metabolic end products. | ^2,73,80^ |
| *Prevotella baroniae* | *Segatella baroniae* | Pigmented anaerobic oral species isolated from human subgingival plaque and associated with chronic periodontitis; characterized as proteolytic | ^2,73,80^ |
| *Prevotella bryantii* | *Segatella bryantii* | Anaerobic ruminal bacterium involved in degradation and fermentation of plant-derived polysaccharides; demonstrated roles in carbohydrate metabolism, succinate production, and adaptation to fiber-rich rumen environments through specialized carbohydrate-active enzymes and polysaccharide utilization systems. | ^1,2,89,90^ |
| *Prevotella buccae* | *Segatella buccae* | Anaerobic oral and respiratory tract-associated bacterium detected in polymicrobial biofilms and chronic airway infections; associated with inflammatory respiratory conditions, antibiotic resistance determinants, and interactions within complex mucosal microbial communities; Metronidazole resistance reported in infection context | ^1,2,91–93^ |
| *Prevotella cerevisiae* | *Segatella cerevisiae* | Anaerobic bacterium isolated from fermented beer waste and characterized by carbohydrate-fermenting capabilities, including utilization of plant-derived substrates and production of succinate and acetate as metabolic end products. | ^2,94^ |
| *Prevotella copri* | *Segatella copri* | Gut-associated anaerobic bacterium frequently associated with high-fiber diets and complex carbohydrate metabolism; identified as a dominant member of non-Western gut microbiomes with strain-level diversity linked to distinct metabolic and inflammatory phenotypes. Reported in studies examining rheumatoid arthritis, glucose metabolism, host immune modulation, polysaccharide utilization systems, and microbiome-associated disease variability. | ^1,2,83,95–103^ |
| *Prevotella hominis* | *Segatella hominis* | Anaerobic human fecal isolate that produces succinic and acetic acids and has been discussed as a gut-associated Segatella species relevant to intestinal ecology and gut barrier function. | ^2,104^ |
| *Prevotella maculosa* | *Segatella maculosa* | Anaerobic Gram-negative bacterium originally isolated from the human oral cavity and described as a novel oral Prevotella species based on phenotypic and 16S rRNA analyses. A later forensic saliva study identified P. maculosa DNA in highly degraded oral samples using oral bacterial DNA profiling approaches. Subsequent phylogenomic reclassification reassigned the species to the genus Segatella. | ^1,2,6,105,106^ |
| *Prevotella oris* | *Segatella oris* | Originally described as Bacteroides oris from human periodontitis and other human infections, including oral infections. Later clinical reports identified P. oris as a cause of bacteremia and sepsis originating from dentoalveolar abscesses. A subsequent phylogenomic re-evaluation of the genus reassigned the species to Segatella based on genomic distinctiveness from retained Prevotella species. | ^1,2,6,107,108^ |
| *Prevotella oulorum* | *Segatella oulorum* | Originally described as Bacteroides oulorum, a nonpigmented saccharolytic anaerobic bacterium isolated from the human oral cavity. The species was later transferred to the genus Prevotella during reclassification of related oral Bacteroides species. A subsequent phylogenomic analysis reassigned the species to the genus Segatella based on genomic and phylogenetic distinctiveness from retained Prevotella species. | ^1,2,6,109^ |
| *Prevotella paludivivens* | *Segatella paludivivens* | Strictly anaerobic Gram-negative bacterium isolated from plant residue and rice roots in irrigated rice-field soil; characterized as a hemicellulose-degrading species capable of utilizing xylan and other plant-derived polysaccharides. A later phylogenomic analysis reassigned the species to the genus Segatella. | ^1,2,6,31^ |
| *Prevotella salivae* | *Segatella salivae* | Anaerobic bacterium isolated from the human oral cavity and described as a novel species using phenotypic and 16S rRNA analyses alongside Prevotella shahii. A later phylogenomic re-evaluation of the genus reassigned the species to Segatella. | ^1,2,6,76^ |
| *Prevotella brevis* | *Xylanibacter brevis* | Anaerobic ruminal bacterium associated with degradation of hemicellulosic plant polysaccharides; characterized by xylanolytic activity and carbohydrate fermentation capabilities supporting a role in fiber digestion within the rumen microbiome. | ^1,87,110^ |
| *Prevotella muris* | *Xylanibacter muris* | Gut-associated anaerobic bacterium identified in studies showing that colonization with Prevotella species enhanced host susceptibility to mucosal inflammation following gut microbiome perturbation. A later phylogenomic analysis reassigned P. muris to the genus Xylanibacter. | ^6,84^ |
| *Prevotella oryzae* | *Xylanibacter oryzae* | Originally described as Xylanibacter oryzae, a strictly anaerobic, Gram-negative, xylanolytic bacterium isolated from rice-plant residue in flooded rice-field soil in Japan. The species was later reclassified as Prevotella oryzae based on phylogenetic and phenotypic analyses with an emended description of the genus Prevotella. A subsequent phylogenomic re-evaluation reassigned the species back to the genus Xylanibacter. | ^6,111,112^ |
| *Prevotella rara* | *Xylanibacter rara* | Anaerobic Gram-negative bacterium isolated from human feces and characterized as a novel species using culturomics, phenotypic testing, MALDI-TOF MS, and genomic analyses. A later phylogenomic re-evaluation of the genus reassigned the species to Xylanibacter based on genomic distinctiveness from retained Prevotella species. | ^2,6,113^ |
| *Prevotella rodentium* | *Xylanibacter rodentium* | Gut-associated bacterium identified in studies demonstrating that colonization with Prevotella species enhanced susceptibility to mucosal inflammation following gut microbiome perturbation. A later phylogenomic re-evaluation of the genus reassigned P. rodentium to Xylanibacter based on genomic and phylogenetic distinctiveness from retained Prevotella species. | ^2,6,84^ |
| *Prevotella ruminicola* | *Xylanibacter ruminicola* | Succinic acid-producing anaerobic bacterium isolated from the bovine rumen and noted for substantial phenotypic diversity among ruminal isolates. Later phylogenomic analysis reassigned the species to the genus Xylanibacter. | ^1,2,6,114,115^ |

**Supplemental Table 1.** Comprehensive reference table of historically classified *Prevotella* species, including historical nomenclature, current taxonomy, and reported biological attributes compiled from the primary literature. This supplemental table serves as a reference guide for cross-comparing historical and updated taxonomic designations across studies involving members of the family *Prevotellaceae*.

**References:**

1.	Tett, A., Pasolli, E., Masetti, G., Ercolini, D. & Segata, N. Prevotella diversity, niches and interactions with the human host. *Nat Rev Microbiol* **19**, 585–599 (2021).

2.	Wang, S., Zhou, T., Wang, X., Zhao, J. & Wang, X. Bridging the gap: Prevotella/Segatella’s impact on gut barrier function and advanced cultivation strategies to realize the uses in gut health. *Gut Microbes* **18**, 2638001 (2026).

3.	Filardo, S. *et al.* Selected Immunological Mediators and Cervical Microbial Signatures in Women with Chlamydia trachomatis Infection. *mSystems* **4**, e00094-19 (2019).

4.	Sakamoto, M., Suzuki, N. & Okamoto, M. Prevotella aurantiaca sp. nov., isolated from the human oral cavity. *Int J Syst Evol Microbiol* **60**, 500–503 (2010).

5.	Fteita, D. *et al.* Dipeptidyl peptidase IV and quorum sensing signaling in biofilm-related virulence of Prevotella aurantiaca. *Anaerobe* **48**, 152–159 (2017).

6.	Hitch, T. C. A. *et al.* A taxonomic note on the genus *Prevotella*: Description of four novel genera and emended description of the genera *Hallella* and *Xylanibacter*. *Systematic and Applied Microbiology* **45**, 126354 (2022).

7.	Aroutcheva, A., Ling, Z. & Faro, S. Prevotella bivia as a source of lipopolysaccharide in the vagina. *Anaerobe* **14**, 256–260 (2008).

8.	Gilbert, N. M. *et al.* Gardnerella vaginalis and Prevotella bivia Trigger Distinct and Overlapping Phenotypes in a Mouse Model of Bacterial Vaginosis. *J Infect Dis* **220**, 1099–1108 (2019).

9.	Veloo, A. C. M., Chlebowicz, M., Winter, H. L. J., Bathoorn, D. & Rossen, J. W. A. Three metronidazole-resistant Prevotella bivia strains harbour a mobile element, encoding a novel nim gene, nimK, and an efflux small MDR transporter. *J Antimicrob Chemother* **73**, 2687–2690 (2018).

10.	Buhl, M., Dunlap, C. & Marschal, M. Prevotella brunnea sp. nov., isolated from a wound of a patient. *Int J Syst Evol Microbiol* **69**, 3933–3938 (2019).

11.	Grabner, E. *et al.* Expanding the rumen Prevotella collection: The description of Prevotella communis, sp. nov. of ovine origin. *Syst Appl Microbiol* **46**, 126437 (2023).

12.	Tavana, A. M., Drucker, D. B., Hull, P. S. & Boote, V. Phospholipid molecular species distribution of oral Prevotella corporis clinical isolates. *FEMS Immunol Med Microbiol* **21**, 57–64 (1998).

13.	Pearce, M. A., Devine, D. A., Dixon, R. A. & van Steenbergen, T. J. Genetic heterogeneity in Prevotella intermedia, Prevotella nigrescens, Prevotella corporis and related species isolated from oral and nonoral sites. *Oral Microbiol Immunol* **15**, 89–95 (2000).

14.	Tibi, M. F. *et al.* Modulation of host proteostasis by Prevotella corporis via induction of the heat shock response. *Cell Stress Chaperones* **31**, 100150 (2026).

15.	Ciodaro, F., Gazia, F., Galletti, B. & Galletti, F. Hyperbaric oxygen therapy in a case of cervical abscess extending to anterior mediastinum, with isolation of Prevotella corporis. *BMJ Case Rep* **12**, e229873 (2019).

16.	Cobo, F. *et al.* A rare case of pleural effusion due to Prevotella dentalis. *Anaerobe* **54**, 144–145 (2018).

17.	Willems, A. & Collins, M. D. 16S rRNA gene similarities indicate that Hallella seregens (Moore and Moore) and Mitsuokella dentalis (Haapsalo et al.) are genealogically highly related and are members of the genus Prevotella: emended description of the genus Prevotella (Shah and Collins) and description of Prevotella dentalis comb. nov. *Int J Syst Bacteriol* **45**, 832–836 (1995).

18.	Akhi, R. *et al.* Oral Microbiota Linking Humoral Response, Periodontitis and Atherosclerosis. *J Clin Periodontol* **52**, 1550–1559 (2025).

19.	Takada, K., Hayashi, K., Sato, Y. & Hirasawa, M. Prevotella dentasini sp. nov., a black-pigmented species isolated from the oral cavity of donkeys. *Int J Syst Evol Microbiol* **60**, 1637–1639 (2010).

20.	Sun, B. *et al.* Metagenomic Analysis of Saliva Reveals Disease-Associated Microbiotas in Patients With Periodontitis and Crohn’s Disease-Associated Periodontitis. *Front Cell Infect Microbiol* **11**, 719411 (2021).

21.	Niu, Y. *et al.* Symbiotic relationship between Prevotella denticola and Streptococcus mutans enhances virulence of plaque biofilms. *Arch Oral Biol* **151**, 105714 (2023).

22.	Wu, H. *et al.* Prevotella denticola promotes caries by inducing oral microbial dysbiosis. *BMC Oral Health* **26**, 516 (2026).

23.	Wu, C. C., Johnson, J. L., Moore, W. E. & Moore, L. V. Emended descriptions of Prevotella denticola, Prevotella loescheii, Prevotella veroralis, and Prevotella melaninogenica. *Int J Syst Bacteriol* **42**, 536–541 (1992).

24.	Thomaidis, P. C. *et al.* Sonication assisted microbiological diagnosis of implant-related infection caused by Prevotella disiens and Staphylococcus epidermidis in a patient with cranioplasty. *BMC Res Notes* **8**, 307 (2015).

25.	Nema, S. & Brahmachari, S. Pyonephrosis by Prevotella disiens and Escherichia coli coinfection and secondary peritonitis in an obstructive uropathy patient: A case report and review of the literature. *J Family Med Prim Care* **9**, 1263–1265 (2020).

26.	Dziarski, R., Park, S. Y., Kashyap, D. R., Dowd, S. E. & Gupta, D. Pglyrp-Regulated Gut Microflora Prevotella falsenii, Parabacteroides distasonis and Bacteroides eggerthii Enhance and Alistipes finegoldii Attenuates Colitis in Mice. *PLoS One* **11**, e0146162 (2016).

27.	Yang, C. *et al.* Multi-omics analysis of fecal microbiota transplantation’s impact on functional constipation and comorbid depression and anxiety. *BMC Microbiol* **23**, 389 (2023).

28.	Sakamoto, M. *et al.* Prevotella falsenii sp. nov., a Prevotella intermedia-like organism isolated from monkey dental plaque. *Int J Syst Evol Microbiol* **59**, 319–322 (2009).

29.	Downes, J. & Wade, W. G. Prevotella fusca sp. nov. and Prevotella scopos sp. nov., isolated from the human oral cavity. *Int J Syst Evol Microbiol* **61**, 854–858 (2011).

30.	Ibrahim, M., Subramanian, A. & Anishetty, S. Comparative pan genome analysis of oral Prevotella species implicated in periodontitis. *Funct Integr Genomics* **17**, 513–536 (2017).

31.	Ueki, A., Tonouchi, A., Kaku, N. & Ueki, K. Prevotella herbatica sp. nov., a plant polysaccharide-decomposing anaerobic bacterium isolated from a methanogenic reactor. *Int J Syst Evol Microbiol* **72**, (2022).

32.	Mangalam, A. K. & Murray, J. Microbial monotherapy with Prevotella histicola for patients with multiple sclerosis. *Expert Rev Neurother* **19**, 45–53 (2019).

33.	Zhuang, W.-H. *et al.* Prevotella histicola suppress olfactory bulb damage caused by LPS-induced rhinitis in mice. *Microb Pathog* **206**, 107768 (2025).

34.	Balakrishnan, B. *et al.* Prevotella histicola Protects From Arthritis by Expansion of Allobaculum and Augmenting Butyrate Production in Humanized Mice. *Front Immunol* **12**, 609644 (2021).

35.	Spurgeon, K., Erb-Downward, J. R., Huffnagle, G. B. & Kozik, A. J. Complete genome sequence of Prevotella histicola T05-04. *Microbiol Resour Announc* **14**, e0014425 (2025).

36.	Bertelsen, A., Elborn, S. J. & Schock, B. C. Toll like Receptor signalling by Prevotella histicola activates alternative NF-κB signalling in Cystic Fibrosis bronchial epithelial cells compared to P. aeruginosa. *PLoS One* **15**, e0235803 (2020).

37.	Anani, H. *et al.* Prevotella ihumii sp. nov., a new bacterium isolated from a stool specimen of a healthy woman. *New Microbes New Infect* **32**, 100607 (2019).

38.	Guilhot, E., Lagier, J. C., Raoult, D. & Khelaifia, S. ‘Prevotella ihumii’ sp. nov. and ‘Varibaculum timonense’ sp. nov., two new bacterial species isolated from a fresh human stool specimen. *New Microbes New Infect* **18**, 3–5 (2017).

39.	Bajaj, J. S. *et al.* Distinct gut microbial compositional and functional changes associated with impaired inhibitory control in patients with cirrhosis. *Gut Microbes* **13**, 1953247 (2021).

40.	Jacobsen, T. *et al.* Characterization of Cas12a nucleases reveals diverse PAM profiles between closely-related orthologs. *Nucleic Acids Res* **48**, 5624–5638 (2020).

41.	Buhl, M. E. J., Meier-Kolthoff, J. P. & Marschal, M. Prevotella illustrans sp. nov., derived from human oropharyngeal abscess puncture fluid. *Int J Syst Evol Microbiol* **71**, (2021).

42.	Gmür, R. & Thurnheer, T. Direct quantitative differentiation between Prevotella intermedia and Prevotella nigrescens in clinical specimens. *Microbiology (Reading)* **148**, 1379–1387 (2002).

43.	Teanpaisan, R., Douglas, C. W., Eley, A. R. & Walsh, T. F. Clonality of Porphyromonas gingivalis, Prevotella intermedia and Prevotella nigrescens isolated from periodontally diseased and healthy sites. *J Periodontal Res* **31**, 423–432 (1996).

44.	Mättö, J. *et al.* Role of Porphyromonas gingivalis, Prevotella intermedia, and Prevotella nigrescens in extraoral and some odontogenic infections. *Clin Infect Dis* **25 Suppl 2**, S194-198 (1997).

45.	Zhang, S. *et al.* Current research progress on Prevotella intermedia and associated diseases. *Crit Rev Microbiol* **51**, 545–562 (2025).

46.	Hedberg, M. E. *et al.* Prevotella jejuni sp. nov., isolated from the small intestine of a child with coeliac disease. *Int J Syst Evol Microbiol* **63**, 4218–4223 (2013).

47.	Liu, X. *et al.* Metagenome-genome-wide association studies reveal human genetic impact on the oral microbiome. *Cell Discov* **7**, 117 (2021).

48.	Park, S.-N. *et al.* Prevotella koreensis sp. nov., Isolated from Human Subgingival Dental Plaque of Periodontitis Lesion. *Curr Microbiol* **76**, 1055–1060 (2019).

49.	Shinkai, T. *et al.* Prevotella lacticifex sp. nov., isolated from the rumen of cows. *Int J Syst Evol Microbiol* **72**, (2022).

50.	Takizawa, S. *et al.* Estimation of the Association Between the Rumen Bacterial Abundance and Host Phenotypes of Lactating Dairy Cows Using Structural Equation Modeling. *Anim Sci J* **97**, e70172 (2026).

51.	Kuete Yimagou, E. *et al.* Prevotella marseillensis sp. nov., a new bacterium isolated from a patient with recurrent Clostridium difficile infection. *New Microbes New Infect* **32**, 100606 (2019).

52.	Albright, C. E., Spurgeon, K., Ntamubano, S. & Kozik, A. J. Development of a basal media for carbon utilization assay and analysis of the starch utilization locus in Prevotella melaninogenica. Preprint at https://doi.org/10.1101/2025.07.04.663206 (2025).

53.	El Hafi, B., Jean-Pierre, F. & O’Toole, G. A. Pseudomonas aeruginosa supports the survival of Prevotella melaninogenica in a cystic fibrosis lung polymicrobial community through metabolic cross-feeding. *mBio* **16**, e0159425 (2025).

54.	Chen, J., Xu, Q., Zhang, L., Zhang, D. & Wu, X. Enrichment of prevotella melaninogenica in the lower respiratory tract links to checkpoint inhibitor pneumonitis and radiation pneumonitis. *Front Cell Infect Microbiol* **15**, 1594460 (2025).

55.	Maaloum, M. *et al.* Prevotella merdae sp. nov., a new bacterial species isolated from human faeces. *FEMS Microbiol Lett* **369**, fnac066 (2022).

56.	Kim, Y. *et al.* Fecal microbiota transplantation improves anti-PD-1 inhibitor efficacy in unresectable or metastatic solid cancers refractory to anti-PD-1 inhibitor. *Cell Host Microbe* **32**, 1380-1393.e9 (2024).

57.	Downes, J., Liu, M., Kononen, E. & Wade, W. G. Prevotella micans sp. nov., isolated from the human oral cavity. *Int J Syst Evol Microbiol* **59**, 771–774 (2009).

58.	Sasaki-Imamura, T., Yoshida, Y., Suwabe, K., Yoshimura, F. & Kato, H. Molecular basis of indole production catalyzed by tryptophanase in the genus Prevotella. *FEMS Microbiol Lett* **322**, 51–59 (2011).

59.	Sakamoto, M., Huang, Y., Umeda, M., Ishikawa, I. & Benno, Y. Prevotella multiformis sp. nov., isolated from human subgingival plaque. *Int J Syst Evol Microbiol* **55**, 815–819 (2005).

60.	Könönen, E. *et al.* Phylogenetic characterization and proposal of a new pigmented species to the genus Prevotella: Prevotella pallens sp. nov. *Int J Syst Bacteriol* **48 Pt 1**, 47–51 (1998).

61.	Nograšek, B., Accetto, T., Fanedl, L. & Avguštin, G. Description of a novel pectin-degrading bacterial species Prevotella pectinovora sp. nov., based on its phenotypic and genomic traits. *J Microbiol* **53**, 503–510 (2015).

62.	Afouda, P. *et al.* Noncontiguous finished genome sequence and description of Prevotella phocaeensis sp. nov., a new anaerobic species isolated from human gut infected by Clostridium difficile. *New Microbes New Infect* **15**, 117–127 (2017).

63.	Belkacemi, S. *et al.* Description of Prevotella rectalis sp. nov., a new bacterium isolated from human rectum. *New Microbes New Infect* **36**, 100703 (2020).

64.	Buhl, M. & Marschal, M. Prevotella vespertina sp. nov., isolated from an abscess of a hospital patient. *Int J Syst Evol Microbiol* **70**, 4576–4582 (2020).

65.	Moore, L. V., Johnson, J. L. & Moore, W. E. Descriptions of Prevotella tannerae sp. nov. and Prevotella enoeca sp. nov. from the human gingival crevice and emendation of the description of Prevotella zoogleoformans. *Int J Syst Bacteriol* **44**, 599–602 (1994).

66.	Gupta, V. K., Chaudhari, N. M., Iskepalli, S. & Dutta, C. Divergences in gene repertoire among the reference Prevotella genomes derived from distinct body sites of human. *BMC Genomics* **16**, 153 (2015).

67.	Downes, J., Sutcliffe, I. C., Hofstad, T. & Wade, W. G. Prevotella bergensis sp. nov., isolated from human infections. *Int J Syst Evol Microbiol* **56**, 609–612 (2006).

68.	Buhl, M., Willmann, M., Liese, J., Autenrieth, I. B. & Marschal, M. Prevotella colorans sp. nov., isolated from a human wound. *Int J Syst Evol Microbiol* **66**, 3005–3009 (2016).

69.	Wang, Q. *et al.* Multiomics Analysis Reveals Aberrant Metabolism and Immunity Linked Gut Microbiota with Insomnia. *Microbiol Spectr* **10**, e0099822 (2022).

70.	Dillard, L. R. *et al.* Genome-scale metabolic network reconstruction analysis identifies bacterial vaginosis-associated metabolic interactions. *Nat Commun* **16**, 4768 (2025).

71.	Mehmood, M., Jaffar, N. A., Nazim, M. & Khasawneh, F. A. Bacteremic skin and soft tissue infection caused by Prevotella loescheii. *BMC Infect Dis* **14**, 162 (2014).

72.	Zhang, H., Li, M. & Wang, S. Isolation and identification of hoylesella marshii causing pleural infection: a case report. *BMC Infect Dis* **24**, 677 (2024).

73.	Downes, J., Sutcliffe, I., Tanner, A. C. R. & Wade, W. G. Prevotella marshii sp. nov. and Prevotella baroniae sp. nov., isolated from the human oral cavity. *Int J Syst Evol Microbiol* **55**, 1551–1555 (2005).

74.	Loesche, W. J., Socransky, S. S. & Gibbons, R. J. BACTEROIDES ORALIS, PROPOSED NEW SPECIES ISOLATED FROM THE ORAL CAVITY OF MAN. *J Bacteriol* **88**, 1329–1337 (1964).

75.	Shah, H. N. & Collins, D. M. Prevotella, a new genus to include Bacteroides melaninogenicus and related species formerly classified in the genus Bacteroides. *Int J Syst Bacteriol* **40**, 205–208 (1990).

76.	Sakamoto, M. *et al.* Prevotella shahii sp. nov. and Prevotella salivae sp. nov., isolated from the human oral cavity. *Int J Syst Evol Microbiol* **54**, 877–883 (2004).

77.	Glazunova, O. O., Launay, T., Raoult, D. & Roux, V. Prevotella timonensis sp. nov., isolated from a human breast abscess. *Int J Syst Evol Microbiol* **57**, 883–886 (2007).

78.	Segui-Perez, C. *et al.* Prevotella timonensis degrades the vaginal epithelial glycocalyx through high fucosidase and sialidase activities. *mBio* **15**, e0069124 (2024).

79.	van Smoorenburg, M. Y. *et al.* Vaginal Prevotella timonensis Bacteria Enhance HIV-1 Uptake and Differentially Affect Transmission by Distinct Primary Dendritic Cell Subsets. *Eur J Immunol* **55**, e202451192 (2025).

80.	Alauzet, C. *et al.* Metronidazole resistance in Prevotella spp. and description of a new nim gene in Prevotella baroniae. *Antimicrob Agents Chemother* **54**, 60–64 (2010).

81.	Webb, K. A. *et al.* Genomic diversity and antimicrobial resistance of Prevotella species isolated from chronic lung disease airways. *Microb Genom* **8**, 000754 (2022).

82.	Diop, K. *et al.* Microbial Culturomics Broadens Human Vaginal Flora Diversity: Genome Sequence and Description of Prevotella lascolaii sp. nov. Isolated from a Patient with Bacterial Vaginosis. *OMICS* **22**, 210–222 (2018).

83.	Hayashi, H., Shibata, K., Sakamoto, M., Tomita, S. & Benno, Y. Prevotella copri sp. nov. and Prevotella stercorea sp. nov., isolated from human faeces. *Int J Syst Evol Microbiol* **57**, 941–946 (2007).

84.	Iljazovic, A. *et al.* Perturbation of the gut microbiome by Prevotella spp. enhances host susceptibility to mucosal inflammation. *Mucosal Immunol* **14**, 113–124 (2021).

85.	Maeda, Y. *et al.* Mucosal innate immune activation as the trigger to Prevotella species-induced arthritis in genetically resistant mice. *Cell Rep* **45**, 116755 (2026).

86.	Dolapchiev, L. I. *et al.* Gut Microbiome and Hepatic Transcriptomic Determinants of HCC Development in Mice with Metabolic Dysfunction-Associated Steatohepatitis. *J Hepatocell Carcinoma* **11**, 1891–1905 (2024).

87.	Deusch, S. *et al.* A Structural and Functional Elucidation of the Rumen Microbiome Influenced by Various Diets and Microenvironments. *Front Microbiol* **8**, 1605 (2017).

88.	Walker, N. D., McEwan, N. R. & Wallace, R. J. A pepD-like peptidase from the ruminal bacterium, Prevotella albensis. *FEMS Microbiol Lett* **243**, 399–404 (2005).

89.	Schleicher, L., Fritz, G., Seifert, J. & Steuber, J. Anoxic cell rupture of Prevotella bryantii by high-pressure homogenization protects the Na+-translocating NADH:quinone oxidoreductase from oxidative damage. *Arch Microbiol* **202**, 1263–1266 (2020).

90.	Trautmann, A. *et al.* Short-Chain Fatty Acids Modulate Metabolic Pathways and Membrane Lipids in Prevotella bryantii B14. *Proteomes* **8**, 28 (2020).

91.	Cobo, F., Rodríguez-Granger, J., Sampedro, A. & Navarro-Marí, J. M. Infected breast cyst due to Prevotella buccae resistant to metronidazole. *Anaerobe* **48**, 177–178 (2017).

92.	Sharma, G., Garg, N., Hasan, S., Saffarini, D. & Shirodkar, S. Fumarate and nitrite reduction by Prevotella nigrescens and Prevotella buccae isolated from Chronic Periodontitis patients. *Microb Pathog* **176**, 106022 (2023).

93.	Patel, S., Hanfe, H., Khurana, A. K., Bhadade, A. & Nema, S. Unilateral complicated pleural empyema in a patient with bronchial asthma due to clindamycin-resistant Prevotella buccae. *Arch Clin Cases* **10**, 150–152 (2023).

94.	Nakata, H., Kanda, H., Nakakita, Y., Kaneko, T. & Tsuchiya, Y. Prevotella cerevisiae sp. nov., beer-spoilage obligate anaerobic bacteria isolated from brewery wastewater. *Int J Syst Evol Microbiol* **69**, 1789–1793 (2019).

95.	Abdelsalam, N. A., Hegazy, S. M. & Aziz, R. K. The curious case of Prevotella copri. *Gut Microbes* **15**, 2249152 (2023).

96.	Gu, N. *et al.* Prevotella copri transplantation promotes neurorehabilitation in a mouse model of traumatic brain injury. *J Neuroinflammation* **21**, 147 (2024).

97.	Yang, C. *et al.* Prevotella copri alleviates hyperglycemia and regulates gut microbiota and metabolic profiles in mice. *mSystems* **9**, e0053224 (2024).

98.	Franke, T. & Deppenmeier, U. Physiology and central carbon metabolism of the gut bacterium Prevotella copri. *Mol Microbiol* **109**, 528–540 (2018).

99.	De Simone Carone, L. *et al.* Decoding a Gut Commensal Signal: Structural and Immunological Profiling of Segatella Copri Lipopolysaccharide. *Angew Chem Int Ed Engl* **64**, e202512947 (2025).

100.	Shen, Y. *et al.* Combined Inhibition of the TGF-β1/Smad Pathway by Prevotella copri and Lactobacillus murinus to Reduce Inflammation and Fibrosis in Primary Sclerosing Cholangitis. *Int J Mol Sci* **24**, 11010 (2023).

101.	Hao, Q.-Y. *et al.* Prevotella copri promotes vascular calcification via lipopolysaccharide through activation of NF-κB signaling pathway. *Gut Microbes* **16**, 2351532 (2024).

102.	Li, J. *et al.* A versatile genetic toolbox for Prevotella copri enables studying polysaccharide utilization systems. *EMBO J* **40**, e108287 (2021).

103.	Scher, J. U. *et al.* Expansion of intestinal Prevotella copri correlates with enhanced susceptibility to arthritis. *Elife* **2**, e01202 (2013).

104.	Liou, J.-S. *et al.* Prevotella hominis sp. nov., isolated from human faeces. *Int J Syst Evol Microbiol* **70**, 4767–4773 (2020).

105.	Downes, J., Sutcliffe, I. C., Booth, V. & Wade, W. G. Prevotella maculosa sp. nov., isolated from the human oral cavity. *Int J Syst Evol Microbiol* **57**, 2936–2939 (2007).

106.	Ohta, J. & Sakurada, K. Oral gram-positive bacterial DNA-based identification of saliva from highly degraded samples. *Forensic Sci Int Genet* **42**, 103–112 (2019).

107.	Bein, T., Brem, J. & Schüsselbauer, T. Bacteremia and sepsis due to Prevotella oris from dentoalveolar abscesses. *Intensive Care Med* **29**, 856 (2003).

108.	HOLDEMAN, L. V., MOORE, W. E. C., CHURN, P. J. & JOHNSON, J. L. Bacteroides oris and Bacteroides buccae New Species from Human Periodontitis and Other Human Infections. *International Journal of Systematic and Evolutionary Microbiology* **32**, 125–131 (1982).

109.	Shah, H. N., Collins, M. D., Watabe, J. & Mitsuoka, T. Bacteroides oulorum sp. nov., a Nonpigmented Saccharolytic Species from the Oral Cavity. *International Journal of Systematic and Evolutionary Microbiology* **35**, 193–197 (1985).

110.	Miyazaki, K., Martin, J. C., Marinsek-Logar, R. & Flint, H. J. Degradation and utilization of xylans by the rumen anaerobe Prevotella bryantii (formerly P. ruminicola subsp. brevis) B(1)4. *Anaerobe* **3**, 373–381 (1997).

111.	Sakamoto, M. & Ohkuma, M. Reclassification of Xylanibacter oryzae Ueki et al. 2006 as Prevotella oryzae comb. nov., with an emended description of the genus Prevotella. *Int J Syst Evol Microbiol* **62**, 2637–2642 (2012).

112.	Ueki, A., Akasaka, H., Suzuki, D., Hattori, S. & Ueki, K. Xylanibacter oryzae gen. nov., sp. nov., a novel strictly anaerobic, Gram-negative, xylanolytic bacterium isolated from rice-plant residue in flooded rice-field soil in Japan. *Int J Syst Evol Microbiol* **56**, 2215–2221 (2006).

113.	Efimov, B. A. *et al.* Prevotella rara sp. nov., isolated from human faeces. *Int J Syst Evol Microbiol* **68**, 3818–3825 (2018).

114.	Avgustin, G., Wallace, R. J. & Flint, H. J. Phenotypic diversity among ruminal isolates of Prevotella ruminicola: proposal of Prevotella brevis sp. nov., Prevotella bryantii sp. nov., and Prevotella albensis sp. nov. and redefinition of Prevotella ruminicola. *Int J Syst Bacteriol* **47**, 284–288 (1997).

115.	Bryant, M. P., Small, N., Bouma, C. & Chu, H. Bacteroides ruminicola n. sp. and Succinimonas amylolytica; the new genus and species; species of succinic acid-producing anaerobic bacteria of the bovine rumen. *J Bacteriol* **76**, 15–23 (1958).
